# HIF-1α promotes cellular growth in lymphatic endothelial cells exposed to chronically elevated pulmonary lymph flow

**DOI:** 10.1038/s41598-020-80882-1

**Published:** 2021-01-14

**Authors:** Jason T. Boehme, Catherine J. Morris, Samuel R. Chiacchia, Wenhui Gong, Katherine Y. Wu, Rebecca J. Kameny, Gary W. Raff, Jeffrey R. Fineman, Emin Maltepe, Sanjeev A. Datar

**Affiliations:** 1 Department of Pediatrics, University of California, San Francisco, 513 Parnassus Avenue, HSE1418, Box 1346, San Francisco, CA 94143-1346 USA; 2 Cardiovascular Research Institute, University of California, San Francisco, San Francisco, USA; 3 Department of Surgery, University of California, Davis, Davis, USA

**Keywords:** Congenital heart defects, Cell growth, Transcriptomics

## Abstract

Normal growth and development of lymphatic structures depends on mechanical forces created by accumulating interstitial fluid. However, prolonged exposure to pathologic mechanical stimuli generated by chronically elevated lymph flow results in lymphatic dysfunction. The mechanisms that transduce these mechanical forces are not fully understood. Our objective was to investigate molecular mechanisms that alter the growth and metabolism of isolated lymphatic endothelial cells (LECs) exposed to prolonged pathologically elevated lymph flow in vivo within the anatomic and physiologic context of a large animal model of congenital heart disease with increased pulmonary blood flow using in vitro approaches. To this end, late gestation fetal lambs underwent in utero placement of an aortopulmonary graft (shunt). Four weeks after birth, LECs were isolated and cultured from control and shunt lambs. Redox status and proliferation were quantified, and transcriptional profiling and metabolomic analyses were performed. Shunt LECs exhibited hyperproliferative growth driven by increased levels of Hypoxia Inducible Factor 1α (HIF-1α), along with upregulated expression of known HIF-1α target genes in response to mechanical stimuli and shear stress. Compared to control LECs, shunt LECs exhibited abnormal metabolism including abnormalities of glycolysis, the TCA cycle and aerobic respiration. In conclusion, LECs from lambs exposed in vivo to chronically increased pulmonary lymph flow are hyperproliferative, have enhanced expression of HIF-1α and its target genes, and demonstrate altered central carbon metabolism in vitro. Importantly, these findings suggest provocative therapeutic targets for patients with lymphatic abnormalities.

## Introduction

The accumulation and flow of interstitial fluid is essential for proper development of the lymphatic vasculature^1^. Mechanical forces at the physical interface of the lymph and the vessel initiate cellular transcriptional programs vital to the normal growth and maturation of lymphatic valves, collecting vessels, and mesenteric lymphatics^2–7^. However, exposure of the lymphatic system to abnormal flow and mechanical stimulation can lead to aberrant cellular responses^8–11^ and pathology of the lymph vessels^12^.

The association between abnormal lymphatic function and congenital heart disease (CHD) is well established but the contributing mechanisms are poorly understood^13,14^. Using a clinically relevant ovine model of CHD with markedly elevated pulmonary blood flow (PBF)^15^, we have previously demonstrated that chronic elevation in PBF results in supraphysiologic pulmonary lymph flow that provokes lymphatic dysfunction, including increased baseline tone, augmented contraction, and attenuated endothelium-dependent relaxation of the thoracic duct, and delayed transit kinetics of lymph through the pulmonary lymphatic vasculature. These physiologic abnormalities arise predominantly from lymphatic endothelial dysfunction, as manifest by impaired nitric oxide (NO) signaling both in vitro and *in vivo*^16–19^. Specifically, lymphatic endothelial cells (LECs) from these (shunt) animals exhibit abnormal induction of the shear-responsive transcriptional regulator Krüppel-like Factor 2 (KLF2), which causes increased cellular production of reactive oxygen species (ROS), disrupting NO signaling^19^. This is accompanied by parallel disturbances of NO production in these cells stemming from the disordered homeostasis of nitric oxide synthase (NOS) isoforms^18^.

We believe that these previously characterized changes represent a subset of widespread LEC signaling and transcriptional alterations induced by sustained exposure to pathologically elevated lymph flow. Other groups have performed elegant work beginning to describe mechanotransductive pathways in the lymphatic endothelium that influence growth, development and pathology^1,2,4,5,7–11,20–22^. However, as they have noted, these cellular responses are highly dependent on context such as the nature, magnitude, and duration of the mechanical stimuli investigated^8,9^. Our animal model presents a unique opportunity to study the evolution of LEC responses to prolonged pathologic lymph flow within a relevant anatomic and physiologic context.

Primary LECs isolated from the efferent vessel of the caudal mediastinal lymph node of both shunt and normal control lambs were submitted for next generation sequencing in order to broadly characterize the resulting biologic alterations. This analysis revealed varied cellular responses to environmental stimuli in the shunt LECs, but paradoxically demonstrated activation of pathways associated with hypoxic exposure. While our shunt model results in double the normal levels of pulmonary capillary pressure and pulmonary lymph flow, the lymphatic vascular endothelium is not subjected to abnormalities in environmental O_2_ tension^16^. Cellular adaptations to hypoxia are often diverse and complex, but many aspects of this response flow, either directly or indirectly, from activation of the wide-reaching transcriptional regulator Hypoxia Inducible Factor-1α (HIF-1α)^23^. Although named for its well-characterized induction by environmental hypoxia, HIF-1α has subsequently been shown to respond to a variety of environmental stimuli including shear and stretch^24,25^. We therefore hypothesize that continuous exposure of the lymphatic endothelium to pathologic mechanical forces directly activates HIF-1α via mechanotransductive signaling, leading to widespread alterations in mitochondrial function and cellular growth. To this effect, we assessed changes to cellular transcription and metabolism, redox status, and proliferation in LECs from shunt animals.

## Results

### LECs from shunt animals exhibit broad transcriptional changes

**Figure 1:**
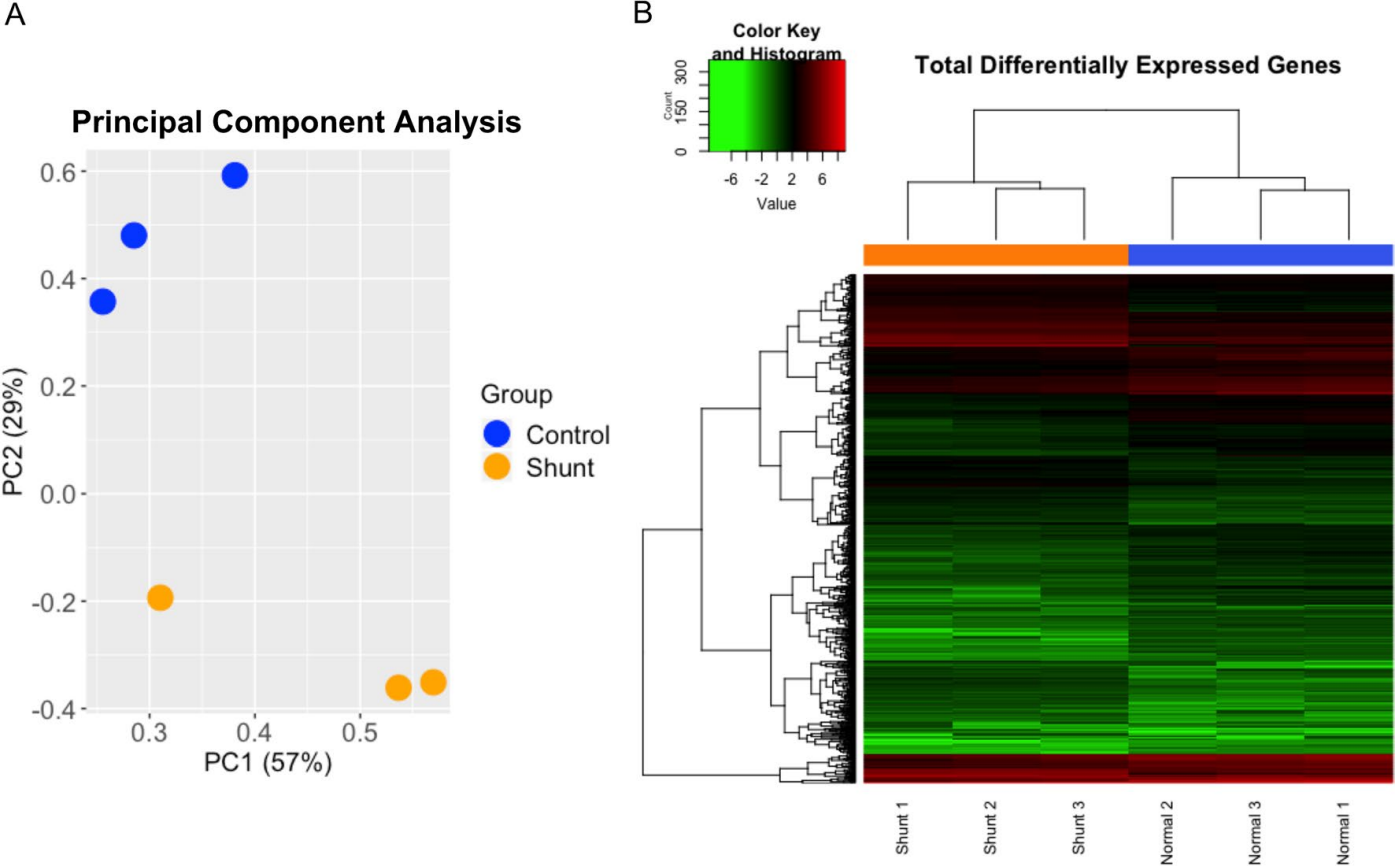
Transcriptional profiling of cultured lymphatic endothelial cells (LECs) isolated from control and shunt lambs. (**A**) Principal component (PC) analysis and (**B**) heat map of RNA-sequencing data demonstrate clustering of differentially expressed genes (DEGs) by model, N = 3 control, 3 shunt.

Primary LEC lines were derived and purified from the efferent vessel of the caudal mediastinal lymph node of shunt and control lambs. RNA seq analysis was performed on primary shunt and control LECs. 4645 differentially expressed transcripts were identified based on a false discovery threshold of < 0.05. In order to optimize pathway enrichment analysis, this pool of differential transcripts was restricted by selecting only those transcripts with an absolute fold change > 2, resulting in ~ 1000 transcripts, of which 834 were mapped onto known human orthologs. 373 of these orthologs were up regulated in shunt LECs, while 461 were down regulated with respect to control cells. Principle component analysis of these 834 differentially expressed genes (DEGs) demonstrated clustering by group, suggesting that the two groups maintain distinct transcriptional profiles (Fig. 1A). This was further supported by unsupervised Euclidean clustering, which demonstrated clear separation between the shunt and control LEC transcriptomes (Fig. 1B). In an effort to further characterize these transcriptional differences, as well as to place them within the context of LEC biology, significantly up-regulated DEGs were submitted to the Gene Ontology (GO) Database for pathway enrichment analysis of biological processes^26,27^. 186 terms were significantly enriched with Fold Enrichment > 3 and FDR < 0.05. Many of these terms referenced redundant or overlapping biological processes, including distal and progressively more proximal categorizations within the same overarching pathway (i.e. serine biosynthesis, serine family amino acid biosynthesis, alpha-amino acid metabolic process, and cellular amino acid biosynthesis). In order to reduce the redundancy of these 186 terms in an unbiased manner, and provide a high-level overview of the pathways impacted, each distal-to-proximal pathway cluster was condensed and reported by only the most proximal term (greatest number of pathway reference genes) that retained pathway fold enrichment > 3. This resulted in 24 distinct terms that are grouped into broad biologic categories and reported in Table 1.

**Table 1:** Pathway enrichment analysis of biologic processes in gene ontology (GO) database.

Go biological process complete	Reference genes in pathway	DEGs matched to pathway	Expected pathway matches	Fold enrichment	+ /−	Raw *p* value	FDR
**Cellular response to environmental stimuli**							
Response to fluid shear stress	34	5	0.61	8.17	+	5.92E−04	4.31E−02
Response to osmotic stress	76	8	1.37	5.85	+	1.20E−04	1.38E−02
Cellular response to oxygen levels	216	13	3.89	3.34	+	2.35E−04	2.34E−02
Response to mechanical stimulus	212	13	3.82	3.41	+	1.98E−04	2.02E−02
**Metabolism and molecular transport**							
Amino acid activation	50	6	0.9	6.67	+	4.53E−04	3.64E−02
Glycosaminoglycan metabolic process	151	10	2.72	3.68	+	6.07E−04	4.38E−02
Cellular amino acid metabolic process	323	21	5.82	3.61	+	9.74E−07	3.28E−04
Small molecule biosynthetic process	578	35	10.41	3.36	+	1.26E−09	1.99E−06
Regulation of lipid metabolic process	397	22	7.15	3.08	+	6.31E−06	1.26E−03
Organic hydroxy compound metabolic process	445	25	8.01	3.12	+	1.16E−06	3.83E−04
Neutral amino acid transport	36	6	0.65	9.26	+	9.01E−05	1.08E−02
L-amino acid transport	58	9	1.04	8.62	+	2.66E−06	7.14E−04
organic acid transport	286	16	5.15	3.11	+	1.04E−04	1.22E−02
**Cellular differentiation and development**							
Regulation of fat cell differentiation	121	11	2.18	5.05	+	2.34E−05	3.50E−03
Regulation of epithelial cell differentiation	138	12	2.48	4.83	+	1.52E−05	2.45E−03
Mesenchymal cell differentiation	151	10	2.72	3.68	+	6.07E−04	4.40E−02
Regulation of muscle cell differentiation	152	10	2.74	3.65	+	6.36E−04	4.51E−02
mammary gland development	132	10	2.38	4.21	+	2.23E−04	2.23E−02
Placenta development	151	11	2.72	4.05	+	1.51E−04	1.63E−02
**Proliferation and apoptosis**							
Negative regulation of vascular smooth muscle cell proliferation	18	4	0.32	12.34	+	5.47E−04	4.08E−02
Negative regulation of apoptotic signaling pathway	226	16	4.07	3.93	+	7.08E−06	1.37E−03
**Response to signalling molecules**							
Response to vitamin D	31	6	0.56	10.75	+	4.30E−05	5.71E−03
Response to purine-containing compound	155	12	2.79	4.3	+	4.41E−05	5.82E−03
Response to steroid hormone	333	22	6	3.67	+	4.16E−07	1.73E−04

### Transcriptional profiles of shunt LECs corroborate widespread activation of hypoxic and mechanotransductive cellular signaling pathways

**Figure 2:**
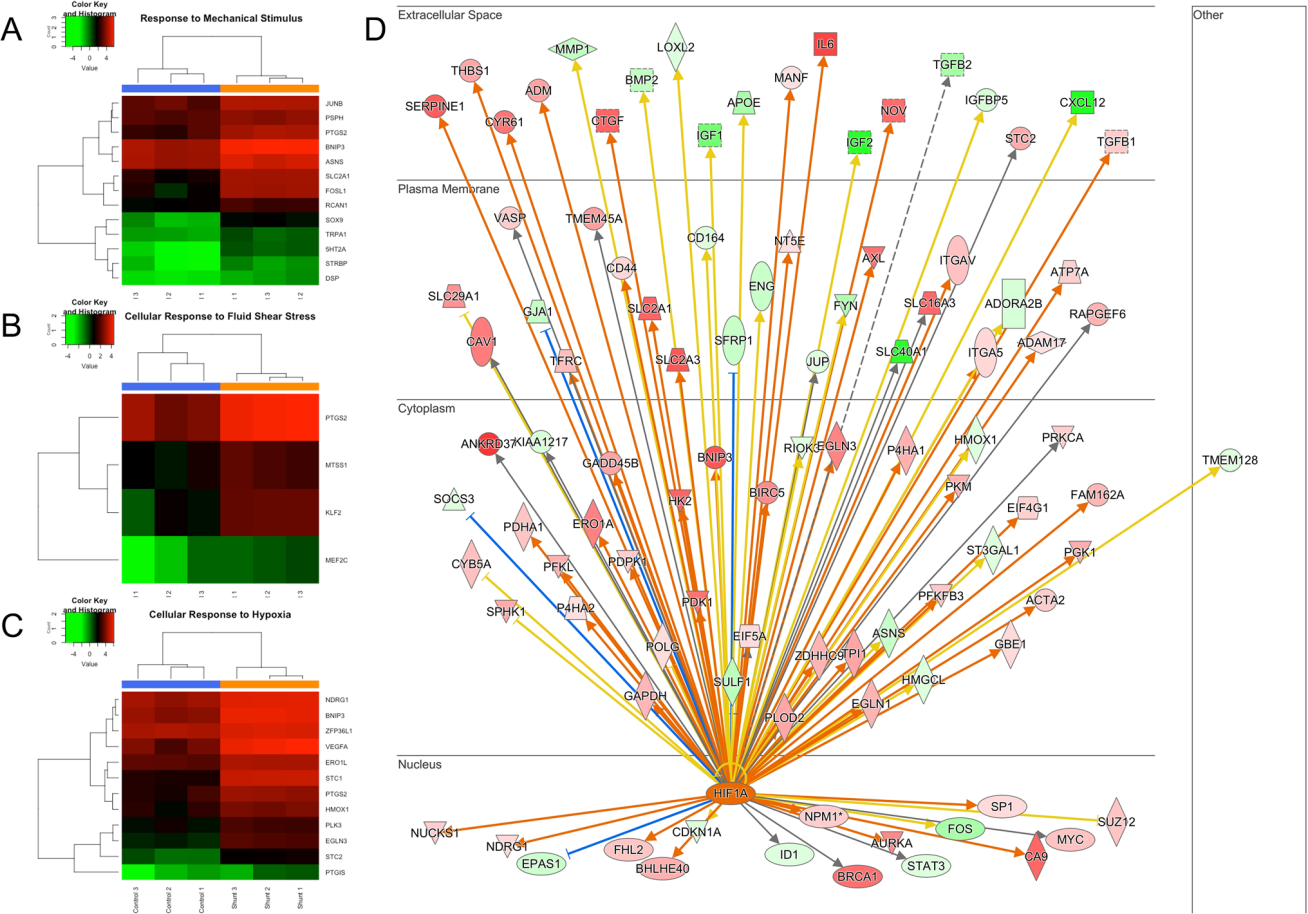
RNA-sequencing analysis. Supervised clustering heat maps demonstrate that upregulated DEGs mapped to processes activated in (**A**) the response to mechanical stimulus, (**B**) the cellular response to fluid shear stress, and (**C**) the cellular response to hypoxia. (**D**) DEGs targeted by HIF-1α in shunt LECs, as predicted by Ingenuity Pathway Analysis upstream regulator modeling. N = 3 control, 3 shunt.

Transcriptional profiling demonstrated that shunt LECs exhibit significant activation of pathways related to environmental stimuli as seen in the GO terms listed in Table 1, including response to fluid shear stress and response to mechanical stimulus (Fig. 2A,B). Additionally, the transcriptional profile of these LECs indicated activation of cellular responses to oxygen levels. Examining the more specific branch terms within this GO pathway, the great majority of genes enriching this pathway were specifically related to the cellular hypoxic response (Fig. 2C). To better correlate these DEGs with more specific upstream regulators, both the entire set of 4645 differentially expressed transcripts, and the more restricted set consisting of DEGs with FC > 2, were evaluated using IPA (QIAGEN Inc.) core analysis algorithm. In addition to standard pathway enrichment algorithms based on the Ingenuity Knowledge Base, IPA upstream regulator analysis predicts likely upstream regulatory elements to explain directional variations in the experimental data set^28^. This analysis corroborated broad transcriptional activation of cellular hypoxic responses and strongly predicted (activation z-score of 3.566, overlap *p* value 3.11 × 10^−8^) the transcription factor hypoxia inducible factor-1α (HIF-1α) as an upstream activating factor in shunt LECs (Fig. 2D).

### Increased rate of proliferation and HIF-1α stabilization in primary LECs from shunt animals

**Figure 3:**
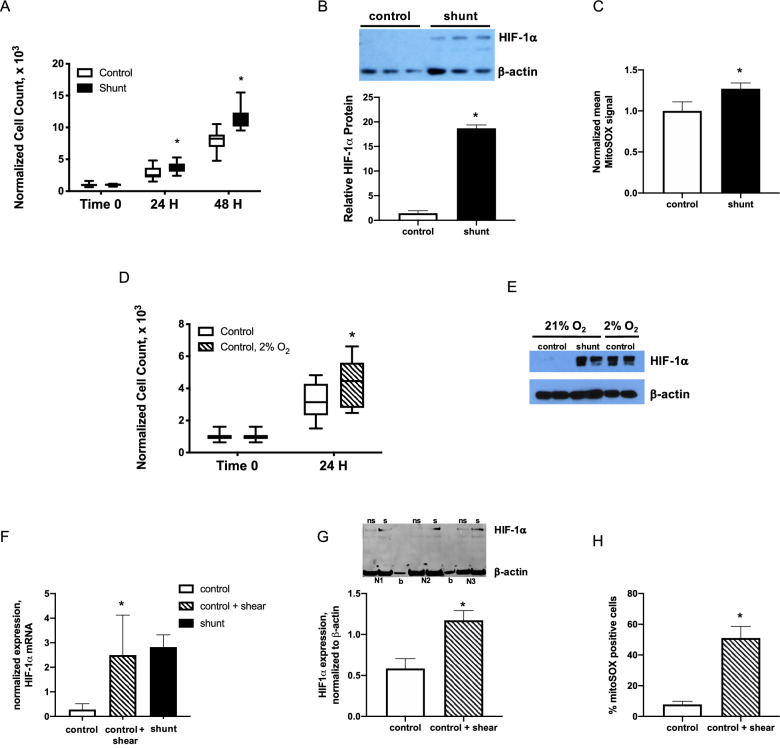
Stabilization of HIF-1α is associated with faster LEC growth and accumulation of mitochondrially-derived reactive oxygen species (ROS). (**A**) shunt LECs proliferate with doubling time 15.2% faster than control LECs, *, *P* < 0.05, N = 3 control, 3 shunt, and (**B**) have 13.1-fold higher expression of HIF-1α, *P* < 0.05, N = 3 control, 3 shunt. NB, bar graph represents HIF-1α protein normalized to β-actin. (**C**) Mitochondrially-derived ROS were increased 1.3-fold in shunt LECs compared to controls, *P* < 0.05. N = 5 control, 5 shunt. NB, bars represent MitoSOX signal normalized to control. (**D**) control LECs cultured in 2% O_2_ proliferate significantly faster than control LECs cultured in 21% O_2_, *, *P* < 0.05; N = 3 control + 21% O_2_, 3 control + 2% O_2_. (**E**) HIF-1α is stabilized in control LECs cultured in 2% O_2_. (**F**–**H**) In control LECs exposed to 0.9 N/m^2^ of shear for 24hrs using a parallel plate flow chamber (ibidi Pump System), (**F**) expression of HIF-1α mRNA is significantly increased compared to levels in non-sheared control LECs, *, *P* < 0.05, N = 3 control no shear, 3 control + shear, 3 shunt; (**G**) similarly, HIF-1α protein is significantly stabilized in sheared (s) control LECs compared with non-sheared (ns) controls, *, *P* < 0.05; N = 3 control no shear, 3 control + shear; and, (**H**) 51% ± 8% sheared control LECs stained positive with MitoSOX compared with 8% ± 2% in non-sheared control LECs, *, *P* < 0.05; N = 3 control no shear, 3 control + shear. In panel F, blank lanes marked by ‘b’. In panels A, B, D, F, G, and H, error bars represent standard deviation. For panels B, E, and G, full-length blots/gels are presented in Supplemental Fig. 6.

Based on results from the RNAseq analyses, the role of HIF-1α in shunt LEC growth and biology was investigated further. Under standard cell culture conditions, primary LECs derived from the shunted animals grew at a significantly increased rate compared to controls (Fig. 3A). Under these same culture conditions, consisting of atmospheric (21%) O_2_ tension, the shunt LECs exhibited greater than ten-fold increased stabilization of HIF-1α compared to controls (Fig. 3B). As proof of principle, control LECs were then cultured under hypoxic conditions (2% O_2_) to induce HIF-1α, resulting in significantly increased HIF-1α stabilization in these cells (Fig. 3E), and also promoting a significantly increased cellular proliferation rate compared to controls cultured under standard conditions (Fig. 3D).

### Mechanical forces promote stabilization of HIF-1α in primary LECs

To validate the principle that mechanical forces can promote stabilization of HIF-1α in the lymphatic endothelium, control LECs were plated on fibronectin-coated slides and exposed for 24hrs to laminar flow at 5 ml/min that generated shear stress of 0.9 N/m^2^, using a parallel plate flow chamber. Compared to the same LEC lines simultaneously plated and incubated on fibronectin-coated slides without corresponding shear, HIF-1α mRNA (Fig. 3F) and protein levels (Fig. 3G) were significantly increased in the shear exposed LECs.

### Increased generation of mitochondrial ROS promotes HIF-1α stabilization and rapid cellular proliferation in shunt LECs

**Figure 4:**
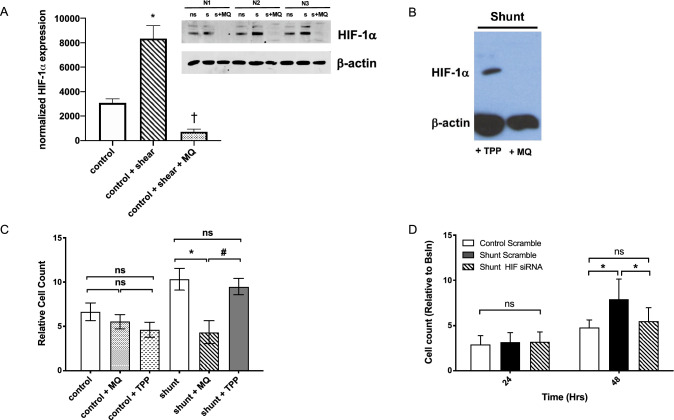
Mitochondrial ROS stabilize HIF-1α and drive HIF-1α-dependent proliferation in shunt LECs. (**A**) HIF-1α protein is significantly stabilized in sheared (s) control LECs compared with non-sheared (ns) controls; treatment of sheared control LECs with a mitochondrially targeted antioxidant mitoquinone (s + MQ) abrogates HIF-1α protein stabilization, *, *P* < 0.05, N = 3 control no shear compared with 3 control + shear; †, *P* < 0.05, N = 3 control + shear compared with 3 control + shear + MQ; (**B**) Treatment of shunt LECs with MQ abrogates HIF-1α expression but does not with triphenylphosphonium (TPP), a lipophilic cationic control; NB, each lane is a pooled sample of 3 distinct shunt LEC lines. (**C**) At 72hrs, treatment of shunt LECs with MQ (angled line bar) slows proliferation to the level observed in control LECs, *P* > 0.05, whether untreated, + MQ, or + TPP (white, speckled, and inverted caret bars, respectively); *, *P* < 0.05 compared to untreated shunt LECs (black bar). Treatment of shunt LECs with TPP (solid gray bar) does not change proliferation compared to untreated shunt LECs (black bar), and remains significantly faster than control LECs or shunt LECs treated with MQ, #, *P* < 0.05. N = 3 control, 3 shunt, for each condition: untreated, + TPP, and + MQ. NB, cell counts normalized to baseline 24 hr timepoint. (**D**) Knockdown of HIF-1α in shunt LECs (angled line bars) slows proliferation to levels observed in scramble treated controls (white bars), *, *P* < 0.05 comparing scramble treated control and shunt LECs, or scramble treated shunt LECs and siHIF-1α treated shunt LECs; N = 3 control + scr, 3 shunt + scr, 3 shunt + siHIF-1α. In panels A, C and D, error bars represent standard deviation. For panels A and B, full-length blots/gels are presented in Supplemental Fig. 6.

LECs from shunt animals have previously been shown to exhibit abnormal cellular redox signaling^18, 19^. Mitochondrial ROS are important regulators of HIF-1α, though increased and decreased ROS have both been variably reported as stabilizing HIF-1α in differing experimental preparations^29–31^. Investigation of the mitochondria as a source of ROS in the LECs was performed using the targeted mitochondrial superoxide indicator MitoSOX, revealing a 27% increase in mitochondrial ROS production in the shunt LECs compared to controls (Fig. 3C). Similarly, in control LECs exposed to 24 h of laminar flow-mediated shear stress of 0.9 N/m^2^, significantly more cells stained positive for mitochondrial ROS (Fig. 3H, SUP FIG 4). Importantly, treatment of these control LECs with the targeted mitochondrial antioxidant mitoquinone (MQ) during shear exposure abrogated HIF-1α stabilization in these cells (Fig. 4A). Treatment of shunt LECs with MQ also significantly impaired HIF-1α stabilization under atmospheric O_2_ conditions (Fig. 4B). Furthermore, inhibition of HIF-1α with either MQ (Fig. 4C), or by selective knockdown of HIF-1α through siRNA targeted transcriptional silencing (Fig. 4D), significantly slowed the growth of shunt LECs to rates comparable to control LECs.

### HIF-1α is a proximal regulator of biological alterations in LECs from shunted animals

**Figure 5:**
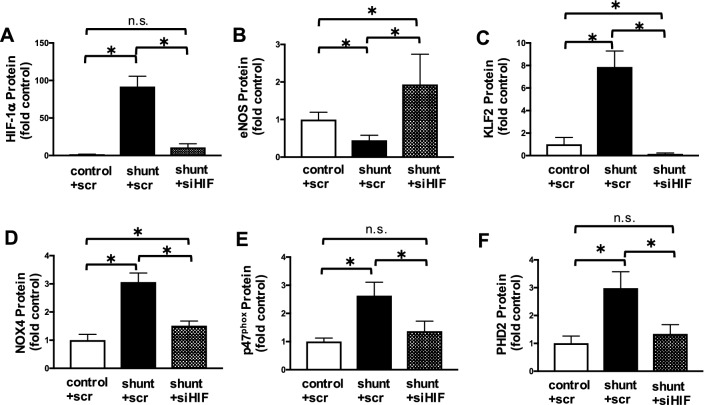
HIF-1α knockdown in shunt LECs. In all panels values are normalized to scramble treated control LECs. (**A**) Treatment with HIF-1α siRNA decreases detectable HIF-1α protein in shunt LECs from 92-fold to 11-fold scramble treated controls. (**B**) In scramble treated shunt LECs, eNOS protein is 0.45-fold scramble treated controls; treatment with HIF-1α siRNA increases eNOS protein in shunt LECs to 1.9-fold scramble treated controls. (**C**) Treatment with HIF-1α siRNA decreases KLF2 protein in shunt LECs from 7.9-fold to 0.14-fold scramble treated controls. (**D**) Treatment with HIF-1α siRNA decreases NOX4 protein in shunt LECs from 3.1-fold to 1.5-fold scramble treated controls, and (**E**) decreases p47^phox^ protein in shunt LECs from 2.6-fold to 1.4-fold scramble treated controls. (**F**) Scramble treated shunt LECs express threefold increased levels of the canonical HIF-1α target PHD2 compared to scramble treated controls, while treatment with HIF-1α siRNA decreases PHD2 protein in shunt LECs to 1.3-fold scramble treated controls. For each panel, *, *P* < 0.05; n = 5 control + scr, 5 shunt + scr, 5 shunt + siHIF-1α, and error bars represent standard deviation.

Important distinct biological alterations of the LECs derived from this ovine shunt model have previously been described, including decreased levels of eNOS and bioavailable NO^18^, increased activity of the transcription regulator Krüppel-like Factor 2 (KLF2)^19^, and increased cellular ROS stemming from both uncoupling of NOS as well as up-regulation of NADPH oxidase (NOX) complexes^18, 19^. To evaluate the impact of HIF-1α stabilization on these previously described biological alterations, HIF-1α was knocked down in shunt LECs using siRNA targeted transcriptional silencing (Fig. 5A). This normalized the expression of canonical HIF-1α target genes, like prolyl hydroxylase (PHD2), to levels comparable to those measured in control LECs (Fig. 5F). Furthermore, knockdown of HIF-1α resulted in a significant increase in the expression of eNOS (Fig. 5B), a significant decrease in the level of KLF2 (Fig. 5C), and decreased expression of NOX enzymes and co-factors (Fig. 5D,E). These results indicate that HIF-1α-dependent signaling can act upstream of KLF2 to regulate LEC biology.

### Shunt LECs exhibit significant alterations in cellular metabolism

**Figure 6:**
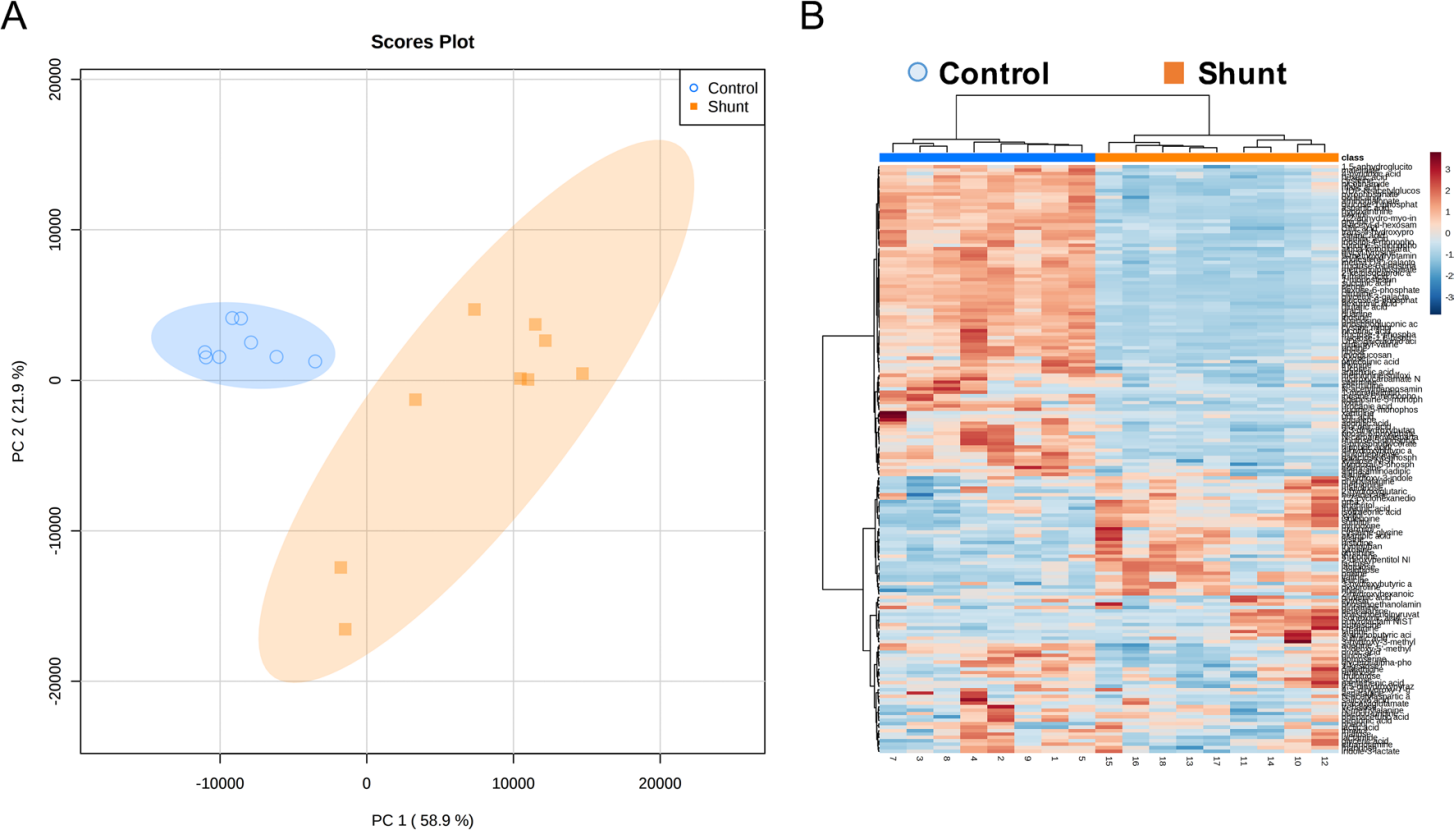
Untargeted metabolomic analysis of cultured LECs isolated from control and shunt lambs, N = 3 control, 3 shunt. (**A**) Principal component analysis and (**B**) heat map of metabolites identified by tandem gas chromatography–mass spectrometry identify differential cellular metabolite concentrations.

The analyses performed by GO and IPA both indicated widespread changes in transcription related to cellular metabolic pathways. Furthermore, HIF-1α is a well-established and prolific regulator of cellular metabolism and it’s activation in varied biological contexts is often associated with metabolic alterations^32^. We performed untargeted metabolomic evaluation of shunt and control LECs using gas chromatography with tandem mass spectrometry to obtain an unbiased overview focused on central carbohydrate and small molecule metabolism. Principal component analysis of all identified metabolites indicated clustering by group, suggesting distinct metabolic profiles (Fig. 6A), and unsupervised Euclidean clustering demonstrated clear separation between the identified shunt and control LEC metabolomes (Fig. 6B). By univariate analysis, 118 identified metabolites were significantly different in the shunt LECs compared to controls using a false discovery rate threshold of < 0.05. Many of these differential metabolites were clustered in identifiable metabolic pathways and classes.

**Figure 7:**
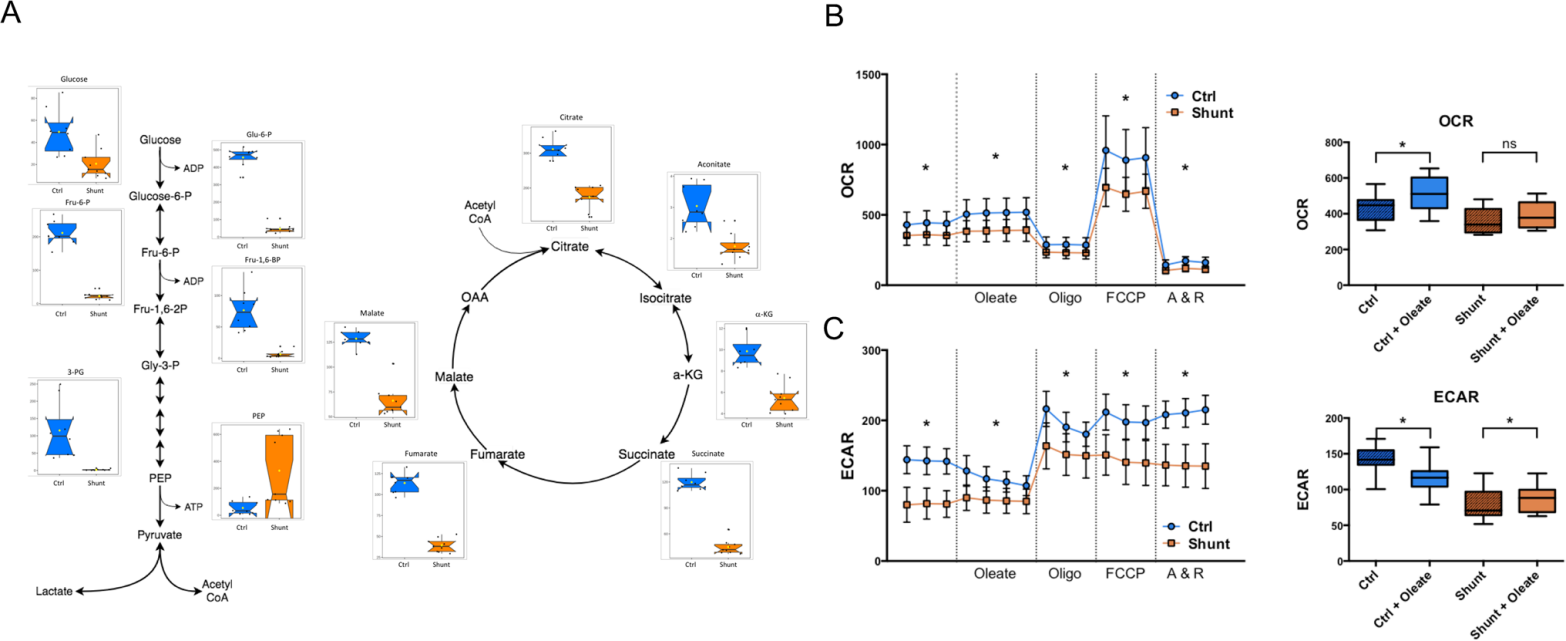
Untargeted metabolomic and extracellular flux analyses demonstrate metabolic abnormalities in shunt LECs. (**A**) Significantly differing metabolites of glycolysis and the TCA cycle in shunt and control LECs by untargeted metabolomic analysis. N = 3 control, 3 shunt. Values expressed as peak intensities with modified vector normalization to the sum of peak heights for all identified metabolites (mTIC) per sample. Data are presented side-by-side as individual data points mapped over notched box and whisker plots with waist at the 50th percentile and edges at the 25th and 75th percentiles respectively. Notch width displays a confidence interval around the median based on the median + / − 1.57 × IQR/sqrt of n. All displayed metabolites exhibit significant differences based on student’s *t* test with *P* < 0.05 adjusted for multiple comparisons using a Bonferroni correction. (**B**) Oxygen consumption rate (OCR, pmol O_2_/min/100,000 cells) and (**C**) Extracellular acidification rate (ECAR mpH/min/100,000 cells) comparisons of shunt (orange squares) and control (blue circles) LECs with N = 2 for each of these representative experiments. For each, repeated measurements are shown for the basal condition and following sequential addition of the metabolites and drugs noted on the x-axis, and separated by dotted vertical lines. For each condition, * denotes a significant difference between pooled values for shunt and control LECs with *P* < 0.05. Also shown is the change in OCR (**B**) and ECAR (**C**) between conditions (basal and following addition of exogenous bioavailable oleic acid) for control and shunt LECs. Pooled values for each condition are presented as box and whisker plots, with * denoting a significant within group difference across conditions with *P* < 0.05.

As seen in Fig. 7A, the intermediate metabolites of glycolysis and the Tricarboxylic Acid (TCA) cycle showed extensive differences in shunt LECs. Almost all identified glycolytic intermediates were significantly reduced in the shunt LECs with the notable exception of phosphoenolpyruvate (PEP), the penultimate metabolite of the pathway, which was significantly elevated. Shunt LECs also exhibited significantly decreased levels of all identified intermediates of the TCA cycle. While this static snapshot indicates significant differences in these pathways, it is not possible to truly assess the pathway rates and activities with this methodology. Low individual metabolite levels for example, may result from either suppression of the pathway upstream, or rapid metabolism downstream.

To better characterize the rate and activity of these pathways, extracellular flux analyses was performed using the Seahorse XF-24 (Agilent)^33^. Given that fatty acids have been strongly implicated as an important energy source for LECs^34^, the standard mitochondrial stress kit was utilized with a modification to include a basal condition without exogenous fatty acid, and a subsequent condition with addition of conjugated oleic acid to the media prior to sequential addition of mitochondrial inhibitors and membrane uncouplers. As seen in Fig. 7B, shunt LECs showed significant repression of oxygen consumption and aerobic metabolism compared to controls. Additionally, shunt LECs did not show the same increase in O_2_ consumption exhibited by control LECs with addition of exogenous fatty acids. The control response matches well with what might be predicted by the Randle cycle, demonstrating increased aerobic metabolism of fatty acids and apparent decrease in glycolysis as the fatty acids become abundant. Interestingly, shunt LECs exhibited significantly lower ECAR than controls, indicating decreased glycolytic lactate production. This result is surprising given the well-established role of HIF-1α as a positive regulator of glycolytic enzymes and activity^35^. Interestingly, addition of exogenous fatty acid demonstrated opposing effects on the ECAR; increasing it in the shunt LECs and decreasing it in the control LECs (Fig. 7C). To assess the role of HIF-1α in these functional metabolic alterations, HIF-1α was suppressed in shunt LECs via siRNA knockdown for 48 h and compared to scramble treated shunt and control LECs in a glucose-only condition using a standard Seahorse mitochondrial stress assay. Shunt LECs treated with HIF-1α siRNA exhibited a partial, but incomplete reversion towards the control LEC phenotype in regard to both oxygen consumption and glycolytic lactate production (SUP FIG 5).

Based on the RNA seq analysis, shunt LECs show broad transcriptional changes impacting amino acid transport and metabolism, and this is corroborated by the metabolic data, which demonstrate significant differences in amino acid levels across a wide range of biosynthetic groups and families (SUP FIG 2). Of particular note is the specific distinctions related to serine and glycine metabolism. Serine is derived from the glycolytic intermediate 3-phosphoglycerate, and is a vital component of biosynthetic pathways via its contributions to one carbon metabolism^36^. The transcriptional data indicated significant upregulation of every enzyme of serine biosynthesis as well as SHMT2, the mitochondrial isoform of the enzyme that converts serine to glycine and mediates the methylation of tetrahydrofolate (SUP Fig. 1A & B) ^36^. Correspondingly the levels of both serine and glycine were significantly lower in shunt compared to control LECs (SUP FIG 1B), in notable contrast to the elevated levels of most other amino acids in these cells (SUP FIG 2).

## Discussion

Vascular endothelial cells are situated at the physical interface of the vessel and its circulating fluid contents. As such, they are at the biologic forefront of vascular responses to fluid dynamics. Mechanoreceptors within the endothelium respond to differential flow patterns and forces to trigger complex cellular and extracellular responses throughout the vessel. These include immediate physiologic changes, along with alterations of cellular growth and biology that may be persistent and lead to eventual vascular remodeling^37,38^. Such mechanotransductive pathways are vital to the growth and differentiation of distinct vascular beds^12^, but may be co-opted by aberrant flow patterns, leading to vascular pathologies such as atherosclerosis^39^ and pulmonary arterial hypertension^40^.

In our ovine model of congenital heart disease with increased pulmonary blood flow and pressure, we have shown that pathologic alterations to pulmonary blood flow result in significantly elevated pulmonary lymph flow and physiologic pulmonary lymphatic dysfunction^15–17^. In this paper we describe wide-reaching biologic alterations in the pulmonary lymphatic endothelial cells (LECs) of this model^15^. In vivo, the pulmonary lymphatic vessels of these animals are subjected to significantly elevated lymph flow on a chronic basis^16^. These changes in flow alter the biomechanical forces to which the lymphatic endothelium is exposed^9^. LECs are extremely attuned to such changes, and have been shown previously to be particularly sensitive to shear, functioning at an optimal biologic homeostasis around a narrowly defined shear-stress “set point” that approximates baseline physiologic exposure^8^. Even small gradations in shear outside this range can provoke widespread cellular responses^8, 9^. In our model of CHD, the LECs are exposed to an increased lymph flow which necessitates increased shear forces, but other mechanical stimuli, such as circumferential or axial stretch^41^, may also be relevant within the lymph vessels in vivo.

In order to broadly characterize the nature of these responses to long-standing elevations in pulmonary lymph flow, cultured LECs from the shunt animals were evaluated by RNAseq, and they revealed a distinct phenotype stemming from the in vivo physiologic alterations. Cellular cascades associated with external environmental stimuli, including mechanical and shear stress, were up-regulated in the shunt LECs, consistent with the known underlying physiology. More surprising was the prominent alteration in pathways related to the cellular hypoxia response, particularly suggesting increased activity of the transcription factor HIF-1α.

HIF-1α was initially recognized as part of the cellular reaction to hypoxic stress, with canonical regulation at the post-translational level mediated via a stabilizing signal arising from the mitochondria in response to hypoxia. There is general agreement that mitochondria modulate HIF-1α stabilization via ROS second messengers, though the exact nature (increased vs decreased) and source of these ROS is subject to continued debate in the literature^30, 31^. It is now recognized that HIF-1α stabilization and activity are mediated through diverse mechanisms, and in response to varied cellular environments and stresses^42^. Indeed, here we have presented evidence that HIF-1α is stabilized in control LECs exposed in vitro to flow-mediated shear (Figs. 3G, 4A). There is precedent in the literature that mechanical forces are included within this group of HIF-1α stabilizing factors. In studies of cultured arterial endothelial cells, separate investigations have demonstrated direct induction of endothelial HIF-1α activity through variations in exposure to shear stress, flow directionality, and stretch^24,25,43^. It is interesting that the nature and magnitude of the provoking forces in these experiments is inconsistent. Though this could be due simply to differences in experimental design and analysis, these results may suggest a response that is variable and dependent on the unique physiologic conditions of distinctive vascular beds. Such context specificity is already described in the fields of both vascular endothelial biology, and HIF-1α biology respectively^12,23^.

HIF-1α is a prolific transcriptional regulator, with over 1000 recognized target genes, but it does not invariably affect these targets in a uniform pattern. Rather, specific subsets of genes are activated according to particular environments and cell types^23^. HIF-1α has long been recognized as an important signal in vascular biology, with essential roles in angiogenesis, vascular growth and development, and cardiovascular disease^44^. The role of HIF-1α in the lymphatic vasculature is less clear, but it has been associated with lymphangiogenesis in the settings of malignancy^45,46^, inflammatory conditions including wound healing^47,48^, and as part of pulmonary lymphatic development^49^. HIF-1α has also been observed to have prominent interactions with canonical regulators of lymphatic identity and development. This includes cooperative binding with the transcription factor GATA2 to activate transcriptional hypoxic response elements^50^, and upstream regulation of VEGFR-3, the receptor for the lymphatic growth factor VEGF-C. The clearest established relationship comes from the work of Han et al. examining the biology of lymphatic malformations, in which they show that HIF-1α activation promotes abnormal LEC growth and migration phenotypes via its effect on VEGFR-3 expression^51^.

In this study we demonstrate significantly increased HIF-1α activity in LECs in response to persistent elevations in lymph flow. Though these cells are not exposed to hypoxic environments in vivo or in vitro, mitochondrial ROS are shown to be a central upstream signal promoting HIF-1α stabilization in response to mechanical forces. While the relationship of mitochondrial ROS and HIF-1α remains contested, our findings of increased mitochondrial ROS in this regard are supported by findings from other experimental models^30^, and the importance of mitochondrial signaling in HIF-1α biology is widely accepted. In line with the work of Han et al., we show that HIF-1α fosters hyperproliferative cellular growth in the shunt LECs, and further demonstrate that interruption of the mitochondrial ROS/HIF-1α signaling cascade can restore a more typical cellular proliferation phenotype. Additionally, our data suggests that HIF-1α may play a proximal role in the mechanotransductive pathways underlying many of the previously described cellular anomalies in LECs chronically exposed to increased flow.

Knocking down HIF-1α in shunt LECs increases the level of eNOS, which has been shown to be pathologically depressed in these cells^18^. eNOS and NO play a vital role in the physiologic functions of vascular endothelium^52^. In the lymphatic vessels, endothelial release of NO is an important regulator of lymphatic pumping and lymph flow^53–55^. We have previously shown that chronic exposure to elevated lymph flow in this model of CHD leads to significantly impaired lymphatic function in vivo due to decreased eNOS expression and deficiency of bioavailable NO^17,18^. Additionally, HIF-1α knockdown suppresses the abnormally elevated levels of KLF2 in the shunt LECs^19^. KLF2 is itself a widely-acting mechanosensitive transcription factor that is induced in vascular endothelial cells by laminar flow-mediated mechanisms^11,47^. In shunt LECs chronically exposed to supraphysiologic shear, KLF2 is abnormally elevated, leading to disruption of PPAR-γ signaling, increased production of ROS by NADPH Oxidase (NOX) complexes, and a further decrease in bioavailable NO^19^. Interestingly, KLF2 has been previously characterized as antagonizing the effects of HIF-1α and promoting its degradation^56, 57^. However, the knockdown effect of HIF-1α on KLF2 seen here suggests a more complex interaction between the two transcription factors.

These results demonstrate a previously unrecognized role of HIF-1α in the lymphatic endothelial response to elevations in lymph flow and mechanical forces. That role includes proximal regulatory control over cellular functions as central as proliferation, redox balance, and NO production. This speaks to a remarkable depth and breadth of the cellular responses mediated by HIF-1α induction. Correspondingly, the IPA analysis of shunt LECs identified greater than 90 differentially expressed genes that vary in accordance with predicted regulation by HIF-1α, many of which are transcription factors, second messengers, or signaling molecules with their own multifaceted downstream effects. Perhaps unsurprisingly given the phenotypic changes relating to cellular proliferation and redox control, the RNA seq analysis also indicates extensive alterations to the cellular metabolic machinery. Metabolism is increasingly recognized as an important governing mechanism in vascular development, growth, and function^58^, with HIF-1α often serving in a prominent regulatory role in these processes^23,44^. HIF-1α has numerous metabolic targets^59^, but is classically characterized by its effects on central carbohydrate metabolism, including up-regulation of glucose transporters and glycolysis, and suppression of mitochondrial oxidation of pyruvate^35,60^.

These canonical hallmarks of HIF-1α are present in the transcriptional profile of shunt LECs, with increased transcription of glucose transporters GLUT1 and GLUT3, the glycolytic enzymes Hexokinase-2 (HK2), Phosphofructokinase (PFK) and Pyruvate Kinase (PK), and the regulatory kinase Pyruvate Dehydrogenase Kinase 1 (PDK1), which restricts the entry of pyruvate into the TCA cycle (SUP FIG 3). Correspondingly, metabolomic analysis of the cells shows striking differences of glycolytic and TCA cycle intermediates, and functional assessments of glycolytic flux and respiration by extracellular flux analysis show significant differences between shunt and control LECs that are at least partially mediated by HIF-1α (Fig. 7 & SUP FIG 5). Interestingly, the physiologic phenotype we find diverges from the classical HIF-1α mediated pattern of aerobic glycolysis. While shunt LECs do exhibit decreased aerobic respiration by extracellular flux analysis, they do not demonstrate the corresponding increase in glycolytic lactate production^61^. This could be explained by specific metabolic adaptations of the LECs, which are known to exhibit greater reliance on fatty acid β-oxidation than other vascular endothelial cells^34^. Indeed, shunt LECs do show a distinctive pattern of alterations to central carbohydrate metabolism with supplementation of exogenous fatty acid. Another possibility suggested by paired findings from the metabolomic and RNAseq data sets indicates potential diversion of the glycolytic metabolite 3-phosphoglycerate (3-PG) through an alternative biosynthetic pathway (detailed in SUP FIG 3). At a regulatory level, transcriptional modifiers in addition to HIF-1α may be having significant interacting effects on cellular metabolism in this model of mechanical stress, as suggested by the broad changes in the transcriptional profile presented. To this effect, the scope of metabolic alterations observed by metabolomic analysis in the shunt LECs extends beyond just the central carbohydrate pathways, implicating diffuse effects on other pathways and cellular programs, such as the widespread increases in amino acid levels across varied synthetic pathway “families” in these cells (SUP FIG 2).

In our efforts to characterize the effects of chronically elevated lymph flow on the lymphatic endothelium, we have uncovered far-reaching alterations of LEC biology that intersect fundamental cellular and vascular phenotypes. HIF-1α is shown to be a novel and pivotal mediator of abnormal proliferation, and is extensively implicated in aberrations of redox control, metabolism, and NO production in these cells. While the immediate signal potentiating HIF-1α stability and activity originates from the mitochondria, the ultimate stimulus is mechanical in nature. Cellular studies have previously shown that the mitochondria of vascular endothelial cells respond to mechanical forces with acute modulation of respiratory activity and ROS production^62,63^, but our findings reveal a comprehensive and sustained response to pathologic hemodynamic conditions that is directly mediated through mitochondrial signaling mechanisms. It remains unclear from this work exactly how mechanical stimuli from the luminal/lymph interface are transmitted to the mitochondria. However, mechanoresponsive ion channels such as Piezo1 and Orai1, along with the VEGFR2/VEGFR3/VE-Cadherin endothelial mechanosensory complex, have all been shown to play important roles in lymphatic growth and development, suggesting any or all of these as potential signal mediators^11,22,64^. Understanding the role of the mitochondria and HIF-1α in the mechanotransductive pathways driving chronic lymphatic vascular abnormalities could lead to innovative therapeutic strategies for children with congenital heart disease and other disorders of lymphatic function.

## Materials and methods

Detailed methods presented in the Online Supplement.

### Chronic model of increased PBF and pulmonary lymph flow

As described^15^, an 8.0 mm vascular graft was anastomosed between the ascending aorta and left pulmonary artery in late gestation fetuses from mixed-breed Western ewes. Four-weeks after spontaneous delivery, hemodynamics were measured and tissue harvested. At the end of each protocol, all lambs were euthanized with a lethal injection of sodium pentobarbital followed by bilateral thoracotomy as described in the NIH Guidelines for the Care and Use of Laboratory Animals. The Institutional Animal Care and Use Committees (IACUC) of the University of California, San Francisco and the University of California, Davis approved all protocols and procedures.

### Isolation and culture of LECs

Primary LEC lines were derived, as described, from an explanted segment of the efferent vessel of the caudal mediastinal lymph node^18^.

### RNAseq

Tissue disruption, mRNA isolation, library synthesis, sequencing, and bioinformatic analysis were conducted by Amaryllis Nucleics (Oakland, CA).

### Baseline and conditional hypoxia proliferation assays

For proliferation studies, three distinct control and shunt LEC lines were plated in triplicate and counted at baseline, 24 h, and 48 h. For hypoxia proliferation assay, LEC lines were counted in triplicate at baseline and 24 h.

### Mitoquinone (MQ) and HIF-1α knockdown proliferation assays

Three distinct LEC lines per group were plated at uniform cell number in triplicate. 24 h after plating, shunt LECs were cultured for the remainder of the experiment in standard culture media with 10umol/L triphenylphosphonium (TPP) or MQ. Post-transcriptional knockdown of gene expression in shunt LECs was performed using custom dicer-substrate siRNA (IDT, IA) per manufacturer’s instructions. Three distinct LEC lines per treatment group were plated in triplicate and counted at baseline, 24 h, and 48 h.

### Untargeted metabolomics analysis

Three distinct shunt and control LEC lines were grown in triplicate (n = 9) and harvested. Extraction and quantification were performed by the UC Davis NIH West Coast Metabolomics Center as described^65^.

### Extracellular flux analysis

Extracellular flux was performed as described^33^ using the Seahorse XF-24 Extracellular Flux Analyzer (Agilent Technologies, Santa Clara, CA) per manufacturer recommendations.

### Applied shear stress on LECs

Three distinct cell lines of confluent control LECs were exposed to 24hrs 0.9 N/m^2^ shear stress using a parallel plate flow chamber (ibidi Pump System, ibidi)^2^. As control, these LEC lines were plated and incubated on fibronectin-coated slides but were not exposed to shear. Cells were either fixed and stained with MitoSOX Red for imaging or were harvested and processed for qPCR and protein determinations as described previously^19^. A slide of each control LEC line that was exposed to shear was co-incubated with 10umol/L MQ; these cells were then processed for protein determination as below.

### Preparation of LEC protein extracts and western blot analysis

Preparation and analysis of protein from LECs was performed as described previously^66^.

### Determination of mitochondrial ROS

Control and shunt LECs were incubated with MitoSOX Red added at a final concentration of 5 mmol/L. Stained LECs were analyzed on a BD LSRFortessa flow cytometer and using FlowJo V10.3.0 software.

### Statistical analysis

As previously described^18,19^, for all experiments not otherwise specified, comparisons in cases of more than two conditions were made using a one-way analysis of variance with a post-hoc Tukey’s multiple comparisons test using GraphPad Prism (version 7.0, GraphPad Software, La Jolla, California). Comparisons between control and shunt conditions were made with the unpaired *t* test. Comparisons between cells in a given line, with or without treatment were made with the paired *t* test. A *p* value of less than 0.05 was considered statistically significant.

## Supplementary Information

Supplementary Information 1.

## Data Availability

The datasets analyzed in the current study are available from the corresponding author on reasonable request.
