# Supplementary material for: HIF-1α promotes cellular growth in lymphatic endothelial cells exposed to chronically elevated pulmonary lymph flow
Source: Sci Rep. 2021 Jan 14;11:1468. doi: 10.1038/s41598-020-80882-1 (PMC7809484; doi:10.1038/s41598-020-80882-1)

# ONLINE SUPPLEMENT

HIF-1α Promotes Cellular Growth in Lymphatic Endothelial Cells Exposed to Chronically Elevated Pulmonary Lymph Flow

Jason T. Boehme[1†], Catherine J. Morris[1†], Samuel R. Chiacchia[1], Wenhui Gong[1], Katherine Y. Wu[1], Rebecca J. Kameny[1], Gary W. Raff[3], Jeffrey R. Fineman[1,2], Emin Maltepe[*,1], and Sanjeev A. Datar[*,1]

1. University of California, San Francisco, Department of Pediatrics
2. University of California, San Francisco, Cardiovascular Research Institute
3. University of California, Davis, Department of Surgery

[†], authors contributed equally to this work

Short title: HIF-1α and cellular growth in lymphatic endothelial cells

*, Corresponding authors:

Sanjeev A. Datar, MD, PhD
sanjeev.datar@ucsf.edu
513 Parnassus Avenue
HSE1418, Box 1346
San Francisco, CA 94143-1346

Emin Maltepe, MD, PhD
emin.maltepe@ucsf.edu
513 Parnassus Avenue
HSE1422, Box 1346
San Francisco, CA 94143-1346

## DETAILED MATERIALS AND METHODS

### *Chronic Model of Increased PBF and Pulmonary Lymph Flow*

As previously described in detail,[1] an 8.0 mm Gore-Tex vascular graft (W.L. Gore and Assoc., Milpitas, CA), was anastomosed between the ascending aorta and left pulmonary artery in anesthetized late gestation fetuses (137-141 days gestation) from mixed-breed Western ewes. Four-weeks after spontaneous delivery, these shunt lambs, and normal, age-matched control lambs were anesthetized, mechanically ventilated, and instrumented to continuously measure hemodynamics and harvest tissue. Pulmonary vascular resistance was calculated using standard formulas. For all shunt lambs, the ratio of pulmonary to systemic blood flow (Qp:Qs) was calculated using the Fick principle. For control lambs, the Qp:Qs was assumed to be 1:1.

At the end of each protocol, all lambs were euthanized with a lethal injection of sodium pentobarbital followed by bilateral thoracotomy as described in the NIH Guidelines for the Care and Use of Laboratory Animals. The Institutional Animal Care and Use Committees (IACUC) of the University of California, San Francisco and the University of California, Davis approved all protocols and procedures.

### *Isolation and Culture of LECs*

After completion of hemodynamic measurements,[2] additional local anesthesia (3ml lidocaine, 2%) was instilled and a right thoracotomy was performed to harvest a segment of the efferent vessel of the caudal mediastinal lymph node.[3,4] From these tissue explants, primary LEC lines were derived as previously described.[3] In total, cell lines were established from five control and five shunt lambs, and expression of the canonical

lymphatic markers LYVE-1 and Prox1 were confirmed for each. For all experiments, LECs between passages 5 and 9 were used. Cells were maintained in (standard) Endothelial Cell Culture Medium (Corning) with 20% fetal bovine serum, penicillin/streptomycin, Fungizone (Thermo Fisher Scientific), and Primocin (InvivoGen) on BioCoat Collagen IV cellware (Corning).

*RNAseq*

Tissue disruption, mRNA isolation, library synthesis, sequencing, and bioinformatic analysis were conducted by Amaryllis Nucleics (Oakland, CA) as previously described in and performed by Song et al., 2018.[5] Cells were lysed in Buffer RLT (Qiagen). mRNA was isolated from cell lysates using the NEBNext Poly(A) mRNA Magnetic Isolation Module (NEB). RNAseq libraries were prepared by using the Full Transcript mode of YourSeq Duet (FT & 3'-DGE) RNAseq Library Kit (Amaryllis Nucleics). A Bioanalyzer 2100 (Agilent, High Sensitivity DNA Kit) was used for library quality control, to determine average library size, and together with concentration data from a Qubit 2.0 Fluorometer (Life Technologies, dsDNA High Sensitivity Assay Kit) to determine individual library molarity and pooled library molarity.[5] A PippinHT (Sage Science) was used to get pooled libraries 250-600 bp in length. These size-selected, pooled libraries were sequenced on a NextSeq 500 (Illumina, High Output v2.5 75 cycle kit) to yield single-read 80 bp reads. As previously described, FASTQ sequence files were preprocessed in two steps. A Python library (clipper.py, https://github.com/mfcovington/clipper) was used to trim off the first 8 nucleotides of each read to remove potential mismatches to the reference sequence caused by annealing of a random hexamer required for library synthesis.

Trimmomatic v0.36 (http://www.usadellab.org/cms/?page=trimmomatic) was used to remove adapter sequences and trim or filter reads based on quality. The parameters used for Trimmomatic were ILLUMINACLIP:TruSeq3-PE-2.fa:2:30:10 LEADING:3 TRAILING:3 SLIDINGWINDOW:4:15 MINLEN:50.[5] Preprocessed reads were mapped to the *Ovis aries* v3.1 genomic reference sequence (ftp://ftp.ensembl.org/pub/release-92/fasta/ovis_aries/dna/Ovis_aries.Oar_ v3.1.dna_sm.toplevel.fa.gz) using HISAT2 (https://ccb.jhu.edu/software/hisat2 /index.shtml). Read counts for each gene in the Ensembl release 92 gene annotation file (ftp://ftp.ensembl.org/pub/release-92/gff3/ovis_aries/Ovis_aries.Oar_v3.1.92.gff3.gz) were calculated using htseq-count (with the -s yes parameter to enforce strand-specific alignment) from the HTSeq Python library (https://academic.oup.com/bioinformatics/article/31/2/166/ 2366196; http://htseq.readthedocs.io/en/master/index.html). Transcript analysis was performed as previously described: the R package edgeR (http://www.bioconductor.org/ packages/release/bioc/html/edgeR.html) was used to identify genes differentially expressed between control and shunt samples.[6] Transcripts were retained for analysis if they had more than two counts per million in at least half of the samples. After normalization factors were calculated and dispersion estimated, genewise exact tests were used to identify expression differences between the two conditions. Differentially expressed genes were then filtered using a false discovery rate (FDR) cutoff of 0.05. FDRs were calculated by adjusting P-values for multiple comparisons using the Benjamini–Hochberg procedure.[5] In order to optimize pathway enrichment analysis, this pool of differential transcripts was then restricted by selecting transcripts with an absolute fold change >2, of which 834 were mapped onto known human orthologs. Principle

component analysis and Euclidean clustering of these 834 differentially expressed genes (DEGs) was performed using R version 3.5.3 (R Core Team 2019).

Significantly up-regulated DEGs were submitted to the Gene Ontology (GO) Database for pathway enrichment analysis of biological processes.[7,8] Both the set of 4645 differentially expressed transcripts, and the more restricted set consisting of DEGs with FC > 2, were evaluated using the Ingenuity Pathway Analysis (IPA; QIAGEN Inc., https://www.qiagenbioinformatics.com/ products/ingenuity-pathway-analysis) core analysis algorithm and upstream regulator analysis.[9]

*Baseline and Conditional Hypoxia Proliferation Assays*

For proliferation studies, shunt and control LEC cell lines (n =3) were counted and plated at uniform cell number onto 24-well BioCoat Collagen IV cellware plates (Corning, NY) in triplicate for each of three time points (baseline, 24hr, 48hr). LECs were maintained in standard media. At sequential 24 hour time points after plating, media and non-adherent cells were aspirated and removed and adherent cells were trypsinized and counted using a desktop coulter based cell counter (Moxi Z, Orflo). For hypoxia proliferation assay, distinct control LEC cell lines (n =3) were counted and plated at uniform cell number onto 24-well BioCoat Collagen IV cellware plates (Corning, NY) in triplicate for each of two time points (baseline, 24hr). Control cells were cultured in standard 5% $CO_2$ cell incubators for the initial 24 hours until the baseline count. Subsequently, the control hypoxia group was maintained at 2% $O_2$ and 5% $CO_2$ in a Heraeus HeraCell 240 variable $O_2$ control cell culture incubator (ThermoFisher Scientific). LECs were maintained in standard culture media and counted at sequential 24 hour time points.

*Mitoquinone (MQ) and HIF-1α Knockdown Proliferation Assays*

3 distinct shunt and control LEC cell lines per treatment group were plated at uniform cell number in triplicate for each of two time points (baseline and 48hr). All LECs were initially maintained in standard cell culture media. 24 hours after plating, media and non-adherent cells were aspirated and removed and adherent cells were trypsinized and counted with Luna-II Automated cell counter (Logos Biosystems, South Korea) to establish baseline time point cell counts. Shunt and control groups were cultured for the remainder of the experiment in standard culture media. Shunt and control+ TPP and shunt and control + MQ groups were cultured for the remainder of the experiment in standard culture media supplemented with TPP (10umol/L) and MQ (10umol/L) respectively. LECs were counted as above for the 48hr time point. For analysis, cell counts at the 48hr time points were normalized to the mean baseline time point cell number by cell line and treatment.

Post-transcriptional knockdown of gene expression in LECs was performed using custom dicer-substrate siRNA (IDT, IA) per manufacturer's instructions. Shunt LECs were incubated with HIF-1α siRNA (sense: 5'rArGrCrUrArUrUrCrArCrCrArArArGrUrUrGrArArUrCrAGA 3', antisense 5' rUrCrUrGrArUrUrCrArArCrUrUrUrGrGrUrGrArArUrArGrCrUrGrA) or scramble siRNA and siTran transfection buffer (Origene, MD) for 48 hours, and control LECs were incubated with scramble siRNA and transfection buffer for 48 hours. Shunt siHIF, shunt scramble, and control scramble LECs were then plated onto 96-well BioCoat Collagen IV cellware plates (Corning, NY). 3 distinct shunt and control LEC cell lines per treatment group were plated at uniform cell number in triplicate for each of three time points

(baseline, 24hr, 48hr). LECs were maintained in standard media. 24 hours after plating, baseline time point cell counts were established and used to normalize LEC counts at the 24hr and 48hr time points as above.

*Untargeted Metabolomics Analysis*

For metabolite quantification, three distinct shunt and control LEC lines were grown in 100mm culture plates in triplicate for each cell line (n = 9). Once cells reached 75-80% confluence, they were harvested as per the UC Davis NIH West Coast Metabolomics Center standard of practice. Media was aspirated and cells were briefly rinsed with cold PBS. Cells were then scraped in cold PBS and pelleted by centrifugation at 4$^{o}$C. Immediately after pelleting, metabolism was quenched by flash freezing in liquid $N_2$. Extraction and quantification were performed by the UC Davis NIH West Coast Metabolomics Center as previously described.[10] Briefly, pellets were thawed on ice and extracted with 1ml of acetonitrile, isopropanol and water in proportions 3:3:2. Samples were mixed and subjected to an additional freeze thaw cycle and mixed again. The samples were centrifuged and the supernatants collected and concentrated to dryness in a cold trap vacuum concentrator. A mixture of internal retention index (RI) markers was prepared using fatty acid methyl esters dissolved in chloroform. Aliquots of this RI mixture were added to the dried extracts, along with a solution of 0.24mol/L methoxyamine hydrochloride in pyridine and the mixture was shaken for 90 min. Then N-methyl-N-trimethylsilyl-trifluoroacetamide with 1% trimethylchlorosilane was added for trimethyl-silylation of acidic protons and shaken for 30 min. The reaction mixtures were transferred to auto-sampler vials and samples were separated on an Agilent 6890 gas

chromatograph using a Restek Rtx-5Sil MS column (30 m length x 0.25 mm internal diameter with 0.25 μm film made of 95% dimethyl/ 5% diphenylpolysiloxane) then analyzed on a Leco Pegasus IV mass spectrometer using electron impact ionization at 70 V. Data pre-processing was performed by the West Coast Metabolomics Center as previously described[10] using ChromaTOF software then exported for further processing using the BinBase algorithm.[11] Signal intensities were reported as peak heights using the unique ion as default, unless an alternative quantification ion was manually set in the BinBase administration software. A quantification report table was produced for all database entries that were positively detected in more than 80% of the samples. Raw data were normalized using a variant of a vector normalization in which each metabolite is normalized to the sum of all peak heights for all identified metabolites (mTIC) in the given sample. Multivariate and univariate analyses were performed using Metaboanalyst 3.0 (http://www.metaboanalyst.ca/).[12] For univariate analyses, t-test is denoted significant for FDR adjusted p-value < 0.05.

*Extracellular flux analysis*

Extracellular flux was performed as previously described[13] using the Seahorse XF-24 Extracellular Flux Analyzer (Agilent Technologies, Santa Clara, CA). Per manufacturer instructions, optimization assays were conducted using both shunt and control LEC lines to establish optimum cellular densities. Experimental assays were then performed at optimal cell density (~30,000 cells/well). Basal metabolism comparisons were performed as per manufacturer recommendations. A set of modified baseline comparisons were performed with addition of Oleic acid conjugated to FFA-Free Bovine serum albumin at

an assay concentration of 100umol/L (Sigma-Aldrich). Mitochondrial stress assay was subsequently performed in the presence of glucose, glutamine and BSA-oleate as per manufacturer's instructions with final well concentrations of Oligomycin 2.5umol/L, FCCP 2umol/L, and Antimycin/Rotenone 0.5umol/L. Media for extracellular flux assays was basic DMEM without NaHOC3, without pyruvate, and without phenol red, supplemented with glutamine 2mmol/L, glucose 5.6mmol/L, and carnitine 0.5mmol/L (Sigma-Aldrich). After re-constitution from powder, media was pH adjusted to 7.40 and sterile filtered. The assay was performed with 4 distinct shunt and control LEC lines that were plated in replicates of 5. Assays were performed in duplicate. Immediately following assays, cells from all wells were counted and averaged per experimental group. OCR and ECAR data for each experimental group were normalized to average cell number for analysis. For siRNA HIF knockdown analysis, post-transcriptional knockdown of gene expression in LECs was performed using custom dicer-substrate siRNA (IDT, IA) per manufacturer's instructions. Shunt LECs were incubated with HIF-1α siRNA (sense: 5'rArGrCrUrArUrUrCrArCrCrArArArGrUrUrGrArArUrCrAGA 3', antisense 5' rUrCrUrGrArUrUrCrArArCrUrUrUrGrGrUrGrArArUrArGrCrUrGrA) or scramble siRNA and siTran transfection buffer (Origene, MD) for 48 hours, and control LECs were incubated with scramble siRNA and transfection buffer for 48 hours. Cells were then trypsinized and re-plated at optimal cell density (~30,000 cells/well) into Seahorse XF 24 cell plates for 24 hours. Basal metabolism and mitochondrial stress test assay were performed as per manufacturer recommendations with final well concentrations of Oligomycin 2.5umol/L, FCCP 2umol/L, and Antimycin/Rotenone 0.5umol/L. Media for extracellular flux assays was basic DMEM without NaHOC3, without pyruvate, and

without phenol red, supplemented with glucose 5.6mmol/L (Sigma-Aldrich) and pH adjusted to 7.40. The assay was performed with 2 distinct shunt and control LEC lines that were plated in replicates of 5. Assays were performed in duplicate. Immediately following assays, cells from all wells were counted and averaged per experimental group. OCR and ECAR data for each experimental group were normalized to average cell number for analysis. To normalize across plates, all individual values of OCR and ECAR were subsequently normalized relative to the average basal measurement for all control cells per plate.

*Applied Shear Stress on LECs*

Three distinct cell lines of confluent control LECs on μ-Slide I (0.4mm) Luer fibronectin-coated slides were exposed, for 24hrs, to laminar flow at 5ml/min that generated shear stress of 0.9 $N/m^2$, using a parallel plate flow chamber (ibidi Pump System, ibidi).[14] As control, these LEC lines were plated and incubated on fibronectin-coated slides but were not exposed to shear. Cells were either fixed in 2% PFA and stained with MitoSOX Red Mitochondrial Superoxide Indicator for imaging or were harvested and processed for qPCR and protein determinations as described previously.[15] A slide of each control LEC line was co-incubated with 10umol/L MQ during the 24hr exposure to shear; these cells were then processed for protein determination as below.

*Preparation of LEC protein Extracts and Western Blot Analysis*

Preparation of protein from LECs for Western blot analysis was performed as previously described.[16] For HIF-1α knockdown, post-transcriptional knockdown of gene expression

in LECs was performed using custom dicer-substrate siRNA as per above. Cell lysates from 5th-9th passage LECs were used. For Western blot analysis, 20mg total protein was separated by 10% SDS-PAGE and transferred to a polyvinylidene difluoride membrane (Millipore Corp, USA). The membranes were then blocked with 5% nonfat dried milk in 130mmol/L NaCl and 25mmol/L Tris (TBS, pH 7.5) for 1h at room temperature and subsequently exposed to primary antibodies against HIF-1α, KLF2, eNOS, p47$^{phox}$ (Santa Cruz Biotechnology, Inc), and NOX4 , PHD2, and β-actin (Abcam); the last of which served as a loading control. Following incubation with the appropriate horseradish peroxidase-conjugated secondary antibodies, chemiluminescence was then used to detect bands (SuperSignal West Pico Chemiluminescent Substrate kit, Pierce Biotechnology, Rockford, IL). Densitometry was performed using a public domain Java image processing program, Image J (NIH Image).

*Determination of Mitochondrial ROS*

Mitochondrial ROS levels were determined using MitoSOX Red Mitochondrial Superoxide Indicator (Life Technologies, Carlsbad, CA). Control and shunt LECs were grown to 70% confluency then incubated at 37°C for 10 minutes with MitoSOX Red added at a final concentration of 5umol/L. Stained cells were then washed three times in PBS, trypsinized (0.25% Trypsin EDTA) for 5 minutes, washed twice more in PBS, and resuspended in PBS for analysis. LECs were passed through a 40µm filter and immediately analyzed on a BD LSRFortessa flow cytometer using FACSDiva software. MitoSOX dye was excited using a 488nm blue, and emissions were measured in the 515 to 545nm range. Data were analyzed using FlowJo V10.3.0 software. To measure the effect of flow-mediated

shear on accumulation of mitochondrial ROS in control LECs, three distinct cell lines of confluent control LECs on μ-Slide I (0.4mm) Luer fibronectin-coated slides were exposed, for 24hrs, to laminar flow at 5ml/min that generated shear stress of 0.9 $N/m^2$, using a parallel plate flow chamber (ibidi Pump System, ibidi)[14]. As a control, these LEC lines were plated and incubated on fibronectin-coated slides but were not exposed to shear. Cells were washed twice in PBS, fixed in 2% PFA, rewashed twice in PBS, and then incubated in media containing MitoSOX at a concentration of 5umol/L for 20min in the dark at 37°C. A Leica DM5000 B fluorescence microscope equipped with a DMC 2900 camera was used for acquisition of fluorescent images using Leica Application Suite Advanced Fluorescence v 4.0 software. For each slide of 3 control LEC lines with and without shear, two to three 633ms exposure images were taken at 10x magnification. Using Image J software, the number of MitoSOX Red positive fluorescent cells were counted across these images and scored as a percentage of the total number of cells counted per slide (400-500 cells for each cell line).

SUPPLEMENTAL FIGURE LEGENDS

**Supplemental Figure 1.** Abnormalities of serine biosynthesis in shunt LECs. (A) Supervised clustering heat map of the RNA-sequencing analysis demonstrates upregulated DEGs mapped to serine biosynthesis and metabolism. (B) Significantly differing metabolites of serine and glycine biosynthesis and metabolism in shunt and control LECs by untargeted metabolomic analysis. N = 3 control, 3 shunt. Values expressed as peak intensities with modified vector normalization to the sum of peak heights for all identified metabolites (mTIC) per sample. Data is presented side-by-side as simple bar graphs with error bars corresponding to SEM, and individual data points mapped over notched box and whisker plots with waist at the $50^{th}$ percentile and edges at the $25^{th}$ and $75^{th}$ percentiles respectively. Notch width displays a confidence interval around the median based on the median +/- 1.57 x IQR/sqrt of n. All displayed metabolites exhibit significant differences based on student's t-test with $P < 0.05$ adjusted for multiple comparisons using a Bonferroni correction. While almost all glycolytic metabolites are significantly reduced in shunt LECs compared to controls (FIG 7A), there is a striking reversal of this pattern for the metabolite PEP, suggesting a possible downstream regulatory blockage, with the subsequent and rate-limiting enzyme PK seeming a likely candidate. Such a bottleneck might be expected to result in accumulation of the upstream metabolite 3-phosphoglycerate (3-PG) as well, but this is not observed (FIG 7B). Based on the RNAseq results, we believe that this is due to diversion and metabolism of 3-PG through the serine biosynthetic pathway. Notably, every enzyme mediating the conversion of 3-PG to serine, and subsequently to glycine, is significantly up-regulated in shunt LECs, and the levels of serine and glycine are also significantly

decreased in these cells. This is in striking contrast to the more general pattern of increased amino acid levels across varied other synthetic pathway "families" in these cells (SUP FIG 2). Serine biosynthesis is essential to cellular growth and metabolic homeostasis, with serine and glycine serving as key biosynthetic precursors. Serine catabolism by the enzyme SHMT2 provides donor carbons, via tetrahydrofolate, to the wide pool of additive "one carbon" metabolic reactions, while glycine is directly incorporated into porphyrins, glutathione, and nucleotides.[17,18] Perturbations of serine biosynthesis have been shown to exert broad effects on central carbohydrate metabolism, including regulation of glycolysis through modulation of PK activity.[19] This pathway is frequently co-opted in malignancies, at least partially through induction of SHMT2 by HIF-1α, in order to promote cellular growth and attenuate cellular oxidative injury.[20] In shunt LECs this pathway could plausibly be adapted for either, or both of these purposes.

**Supplemental Figure 2.** Abnormalities of amino acid levels in shunt LECs. Significantly differing amino acid levels (grouped by synthetic pathway "family") in shunt and control LECs by untargeted metabolomic analysis. N = 3 control, 3 shunt. Values expressed as peak intensities with modified vector normalization to the sum of peak heights for all identified metabolites (mTIC) per sample. Data is presented side-by-side as simple bar graphs with error bars corresponding to SEM, and individual data points mapped over notched box and whisker plots with waist at the 50$^{th}$ percentile and edges at the 25$^{th}$ and 75$^{th}$ percentiles respectively. Notch width displays a confidence interval around the median based on the median +/- 1.57 x IQR/sqrt of n. All displayed metabolites exhibit

significant differences based on student's t-test with $P < 0.05$ adjusted for multiple comparisons using a Bonferroni correction.

**Supplemental Figure 3.** Abnormalities of HIF-1α regulated glucose transporters, glycolytic enzymes, and metabolic regulatory enzymes in shunt LECs. Significantly differing RNA transcript expression in shunt and control LECs by RNA sequencing analysis. N = 3 control, 3 shunt. (A) Glucose transporter GLUT1. (B) Glucose transporter GLUT 3. (C) Glycolytic enzyme Hexokinase 2. (D) Glycolytic enzyme Phosphofructokinase. (E) Glycolytic enzyme Pyruvate Kinase M. (F) Regulatory enzyme Pyruvate Dehydrogenase Kinase 1. Relative expression values represent normalized transcript read counts. All transcripts exhibit significant differences based on unpaired student's t-test with $p < 0.05$.

**Supplemental Figure 4.** Accumulation of mitochondrial ROS in control LECs exposed to shear. MitoSOX staining of (A) control LECs at baseline and (B) control LECs exposed to 24hrs of laminar flow-mediated shear. Both are 633ms exposure images taken with a 10x objective. There was a positive MitoSOX signal in 51% ± 8% of sheared control LECs compared with 8% ± 2% on non-sheared control LECs.

**Supplemental Figure 5.** siRNA HIF-1α knockdown of shunt LECs promotes reversion to control phenotype with regard to oxygen consumption rate and extracellular acidification by extracellular flux analysis. (A) Normalized Oxygen consumption rate (OCR, pmol

$O_2$/min) and (B) Extracellular acidification rate (ECAR mpH/min) comparisons of siScramble treated shunt (orange squares), siHIF-1α treated shunt (red squares) and siScramble treated control (blue circles) LECs with N=2 for each of these representative experiments. All values are normalized to the average basal levels of OCR and ECAR for siScr control LECs and pooled across experiments. For each, repeated measurements are shown for the basal condition and following sequential addition of the drugs noted on the x-axis, and separated by dotted vertical lines. For each condition, * denotes a significant difference between values for siHIF-1α and siScr shunt LECs, and # denotes a significant difference between values for siHIF-1α shunt and siScr control LECs with $P < 0.05$. Also shown is the difference in pooled basal OCR (A) and ECAR (B) between all groups, presented as box and whisker plots.

**Supplemental Figure 6.** Full-length blots/gels with variable exposures as indicated. (A) Figure 3B; (B) Figure 3E: for purposes of better visualization for the main figure, the HIF-1α bands are taken from the longer exposure, and β-actin bands are taken from the shorter exposure; (C) Figure 3G; (D) Figure 4A: aliquots from the same samples were run on simultaneously run gels. Because of distortions within each gel, for the main figure, the HIF-1α bands are taken from the gel on left, and β-actin bands are taken from the gel on the right; and (E) Figure 4B.

SUPPLEMENTAL REFERENCES

1. Reddy, V. M. *et al.* In utero placement of aortopulmonary shunts: A model of postnatal pulmonary hypertension with increased pulmonary blood flow in lambs. *Circulation* **92**, 606–613 (1995).

2. Datar, S. A. *et al.* Altered reactivity and nitric oxide signaling in the isolated thoracic duct from an ovine model of congenital heart disease with increased pulmonary blood flow. *American Journal of Physiology-Heart and Circulatory Physiology* **306**, H954–H962 (2014).

3. Datar, S. A. *et al.* Disrupted NOS signaling in lymphatic endothelial cells exposed to chronically increased pulmonary lymph flow. *Am. J. Physiol. Circ. Physiol.* **311**, H137–H145 (2016).

4. Staub, N. C. *et al.* Preperation of chronic lung lymph fistula in the sheep. *J Surg Res* **19**, 315–320 (1975).

5. Song, Y. H. *et al.* Molecular basis of flowering under natural long-day conditions in Arabidopsis. *Nat. Plants* **4**, 824–835 (2018).

6. Robinson, M. D., McCarthy, D. J. & Smyth, G. K. edgeR: A Bioconductor package for differential expression analysis of digital gene expression data. *Bioinformatics* **26**, 139–140 (2009).

7. The Gene Ontology Consortium *et al.* Gene Ontology : tool for the unification of biology. *Nat. Genet.* **25**, 25–29 (2011).

8. Carbon, S. *et al.* The Gene Ontology Resource: 20 years and still GOing strong. *Nucleic Acids Res.* **47**, D330–D338 (2019).

9. Krämer, A., Green, J., Pollard, J. & Tugendreich, S. Causal analysis approaches

in ingenuity pathway analysis. *Bioinformatics* **30**, 523–530 (2014).

10. Fiehn, O. *et al.* Quality control for plant metabolomics: Reporting MSI-compliant studies. *Plant J.* **53**, 691–704 (2008).
11. Denkert, C. *et al.* Metabolite profiling of human colon carcinoma - Deregulation of TCA cycle and amino acid turnover. *Mol. Cancer* **7**, 1–15 (2008).
12. Chong, J. & Xia, J. MetaboAnalystR: an R package for flexible and reproducible analysis of metabolomics data. *Bioinformatics* **34**, 4313–4314 (2018).
13. Ferrick, D. A., Neilson, A. & Beeson, C. Advances in measuring cellular bioenergetics using extracellular flux. *Drug Discov. Today* **13**, 268–274 (2008).
14. Sabine, A. *et al.* Mechanotransduction, PROX1, and FOXC2 Cooperate to Control Connexin37 and Calcineurin during Lymphatic-Valve Formation. *Dev. Cell* **22**, 430–445 (2012).
15. Morris, C. J. *et al.* KLF2-mediated disruption of PPAR-γ signaling in lymphatic endothelial cells exposed to chronically increased pulmonary lymph flow. *American Journal of Physiology-Heart and Circulatory Physiology* **315**, H173–H181 (2018).
16. Black, S. M. *et al.* Altered Regulation of the ET-1 Cascade in Lambs with Increased Pulmonary Blood Flow and Pulmonary Hypertension. *Pediatr. Res.* **47**, 97–97 (2000).
17. Mattaini, K. R., Sullivan, M. R. & Vander Heiden, M. G. The importance of serine metabolism in cancer. *J. Cell Biol.* **214**, 249–257 (2016).
18. Wang, W. *et al.* Glycine metabolism in animals and humans: Implications for nutrition and health. *Amino Acids* **45**, 463–477 (2013).

19. Reid, M. A. *et al.* Serine synthesis through PHGDH coordinates nucleotide levels by maintaining central carbon metabolism. *Nat. Commun.* **9**, 1–11 (2018).

20. Lindsten, T. *et al.* Serine Catabolism Regulates Mitochondrial Redox Control during Hypoxia. *Cancer Discov.* **4**, 1406–1417 (2014).

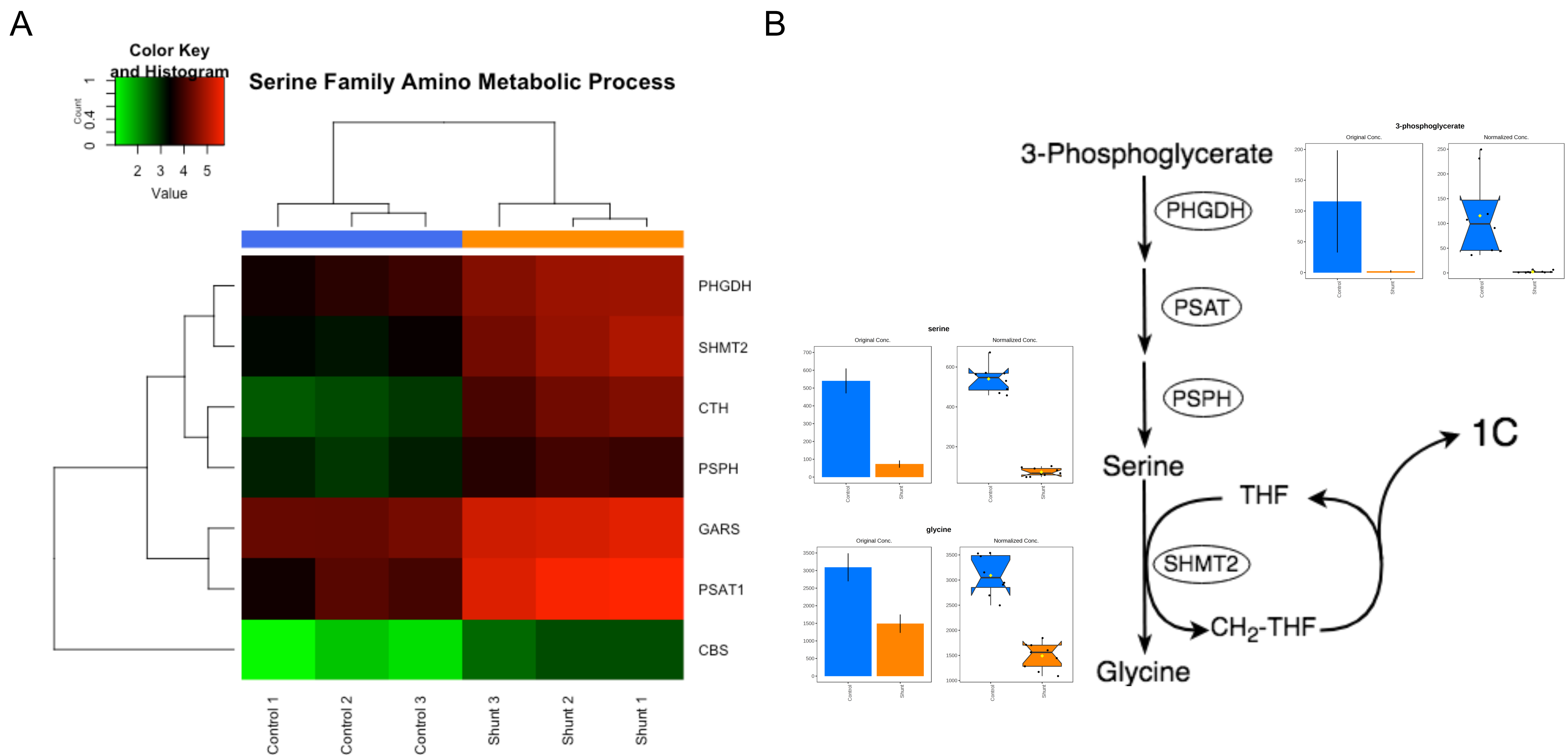
A
Color Key and Histogram
Count
Value
Serine Family Amino Metabolic Process
PHGDH
SHMT2
CTH
PSPH
GARS
PSAT1
CBS
Control 1
Control 2
Control 3
Shunt 3
Shunt 2
Shunt 1
B
3-Phosphoglycerate
PHGDH
PSAT
PSPH
Serine
THF
1C
SHMT2
CH2-THF
Glycine
3-phosphoglycerate
serine
glycine
Original Conc.
Normalized Conc.
Control
Shunt

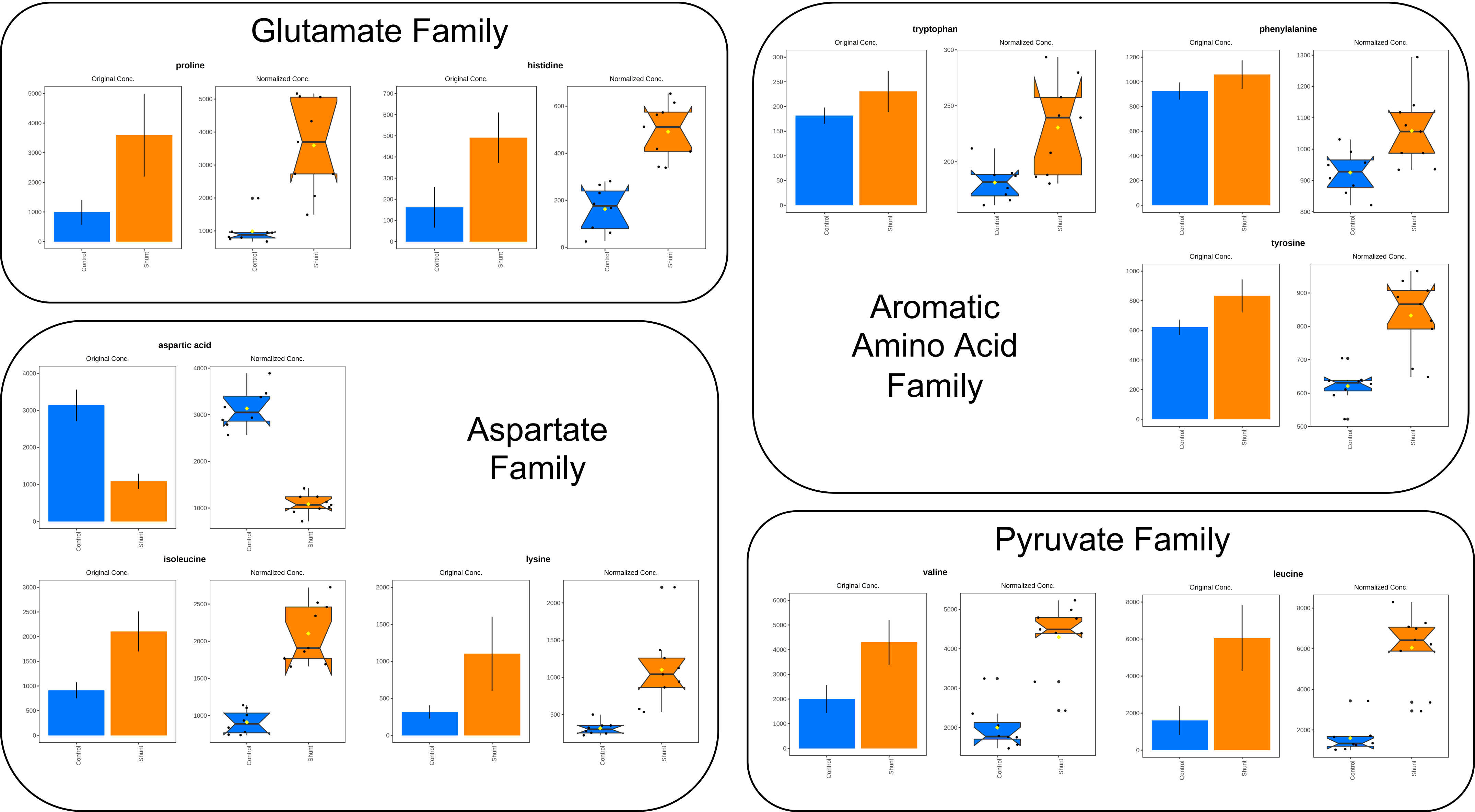

Glutamate Family
proline
Original Conc.
Normalized Conc.
histidine
Original Conc.
Normalized Conc.
Control
Shunt
Aspartate Family
aspartic acid
Original Conc.
Normalized Conc.
isoleucine
Original Conc.
Normalized Conc.
lysine
Original Conc.
Normalized Conc.
Aromatic Amino Acid Family
tryptophan
Original Conc.
Normalized Conc.
phenylalanine
Original Conc.
Normalized Conc.
tyrosine
Original Conc.
Normalized Conc.
Pyruvate Family
valine
Original Conc.
Normalized Conc.
leucine
Original Conc.
Normalized Conc.

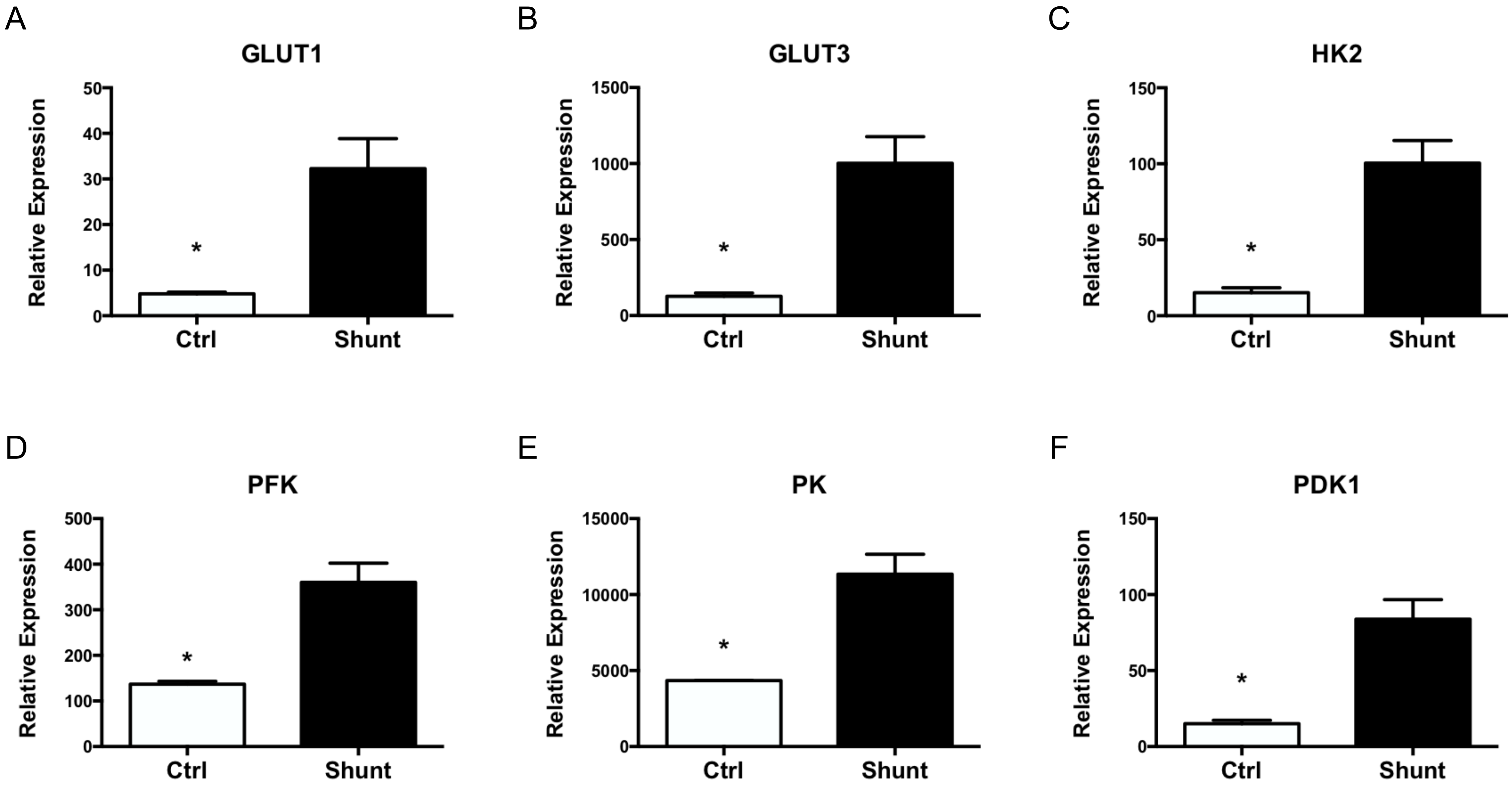
A
GLUT1
B
GLUT3
C
HK2
D
PFK
E
PK
F
PDK1
Relative Expression
Ctrl
Shunt
*
0
10
20
30
40
50
500
1000
1500
100
150
200
300
400
5000
10000
15000

A B

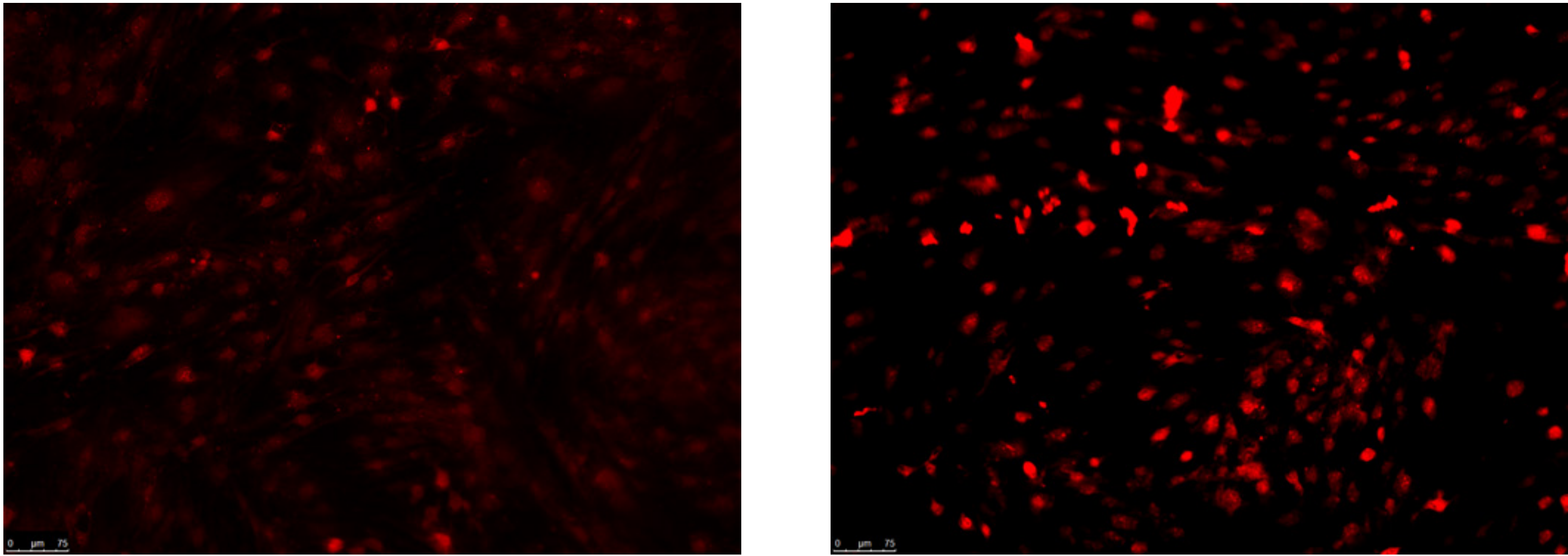


control LECs, no shear

control LECs, + shear

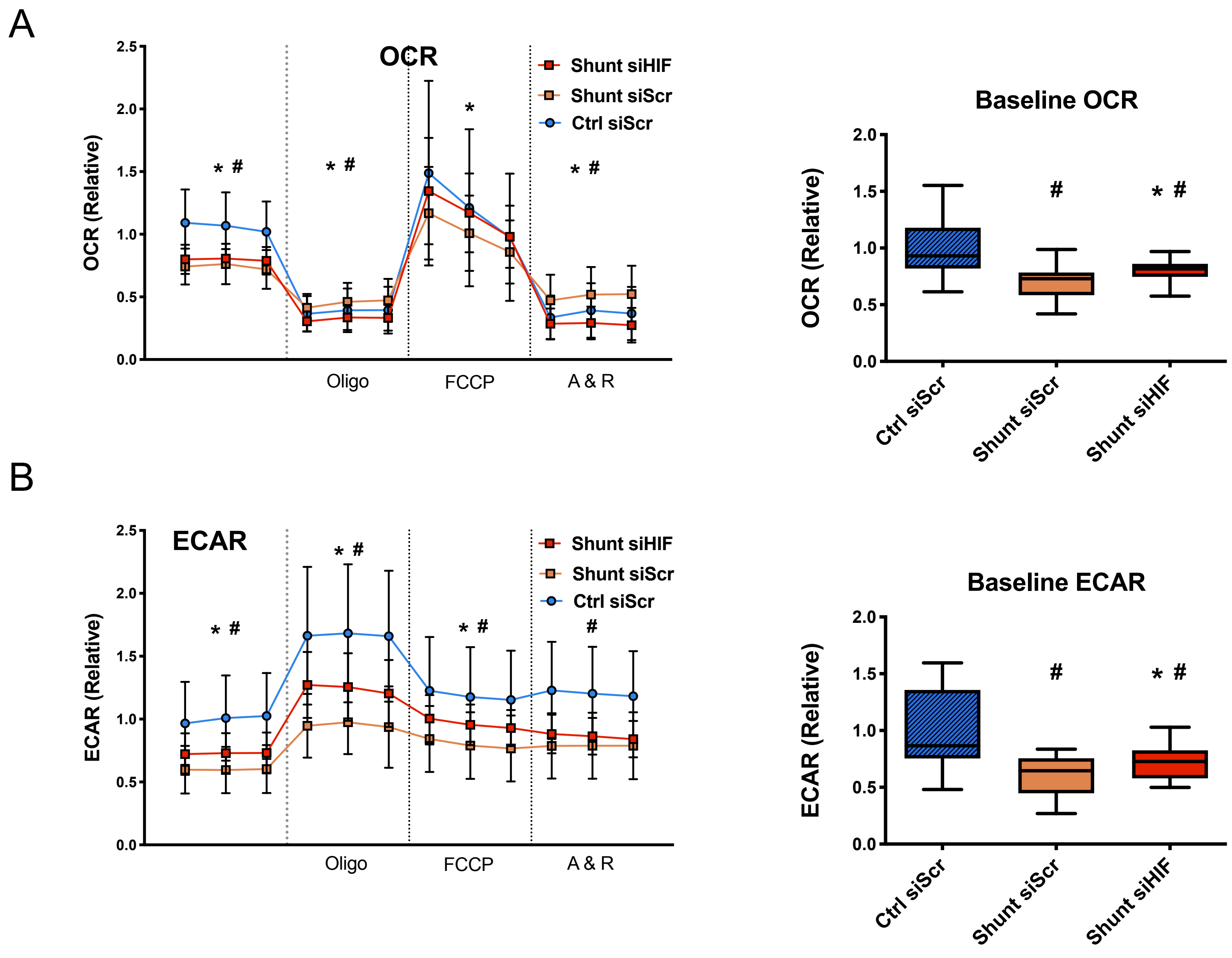
A
OCR
OCR (Relative)
2.5
2.0
1.5
1.0
0.5
0.0
* #
* #
*
* #
Oligo
FCCP
A & R
Shunt siHIF
Shunt siScr
Ctrl siScr
Baseline OCR
OCR (Relative)
#
* #
Ctrl siScr
Shunt siScr
Shunt siHIF
B
ECAR
ECAR (Relative)
* #
* #
* #
#
Oligo
FCCP
A & R
Shunt siHIF
Shunt siScr
Ctrl siScr
Baseline ECAR
ECAR (Relative)
#
* #
Ctrl siScr
Shunt siScr
Shunt siHIF

A

Figure 3B, uncropped blot for figure

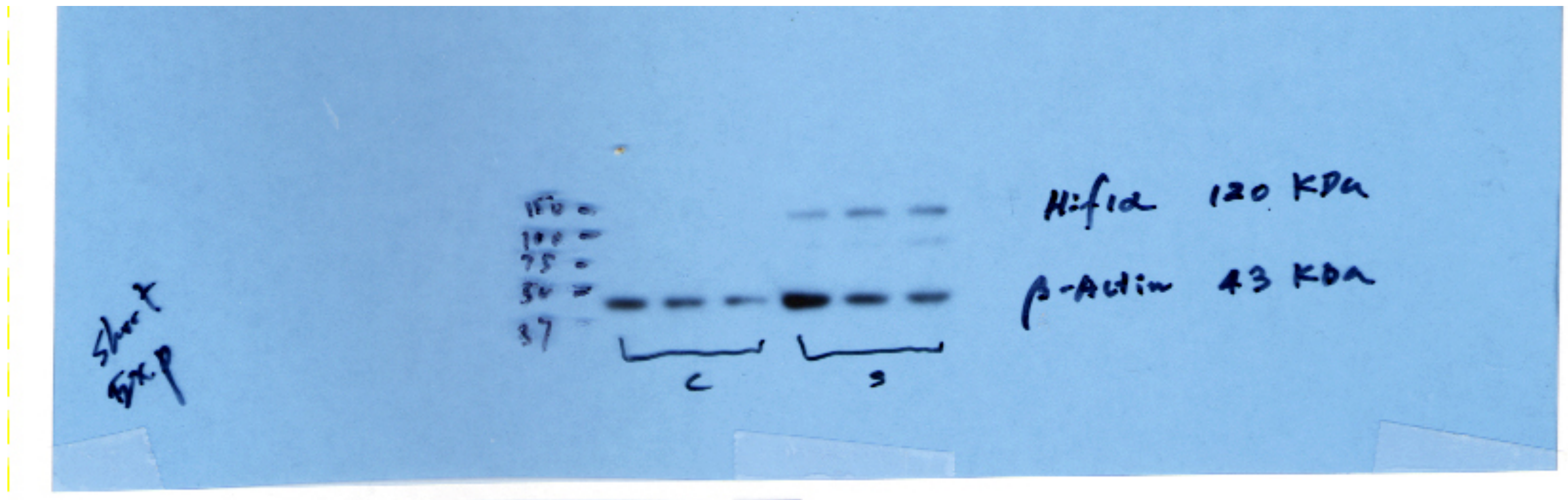


Figure 3B, longer exposure

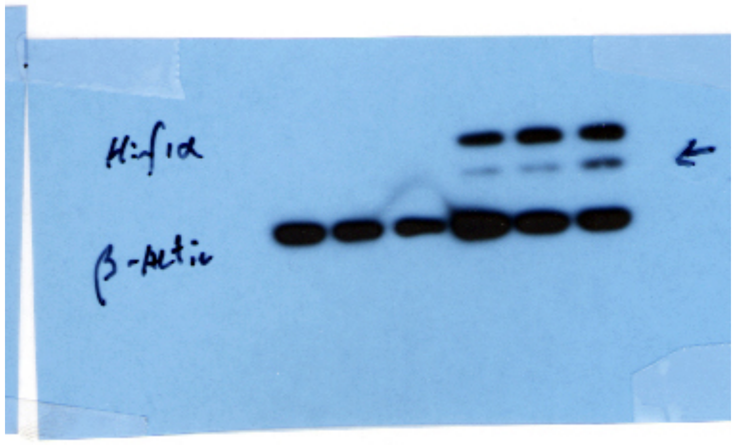


B

Figure 3E, uncropped blot for figure, different exposures

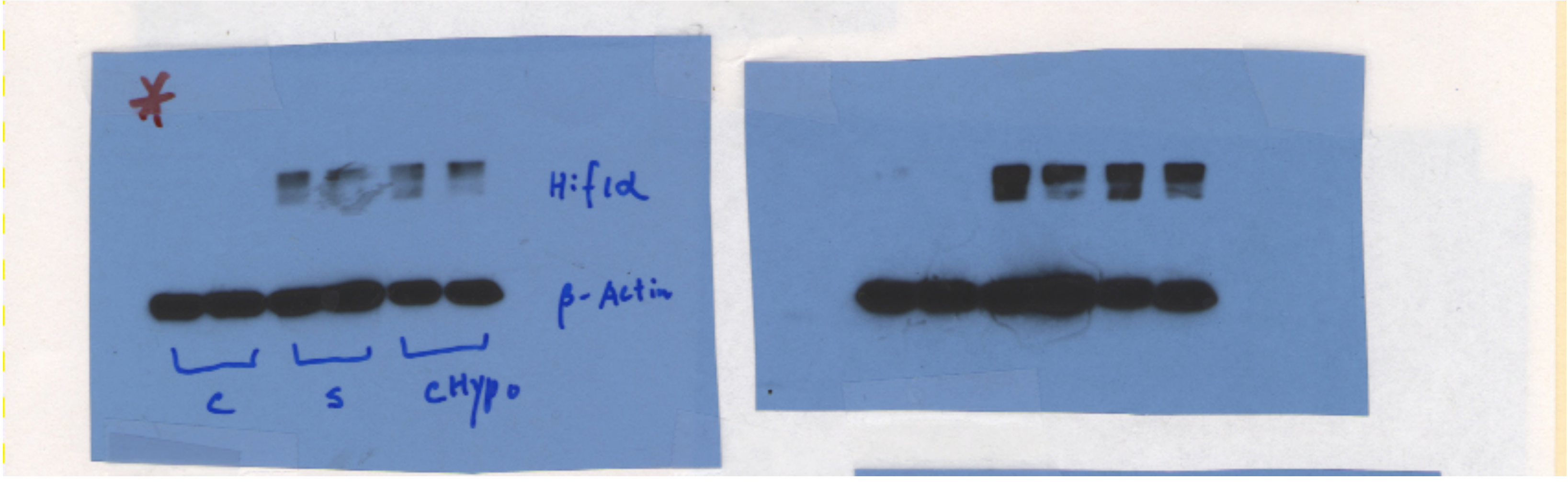


Figure 3E, different gel

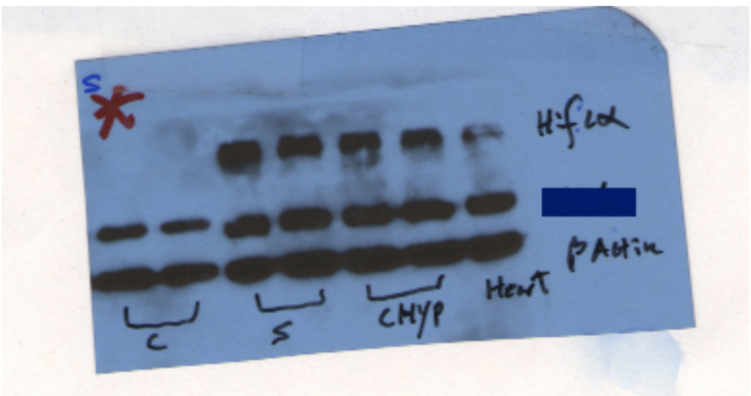


Figure 3E, another different gel

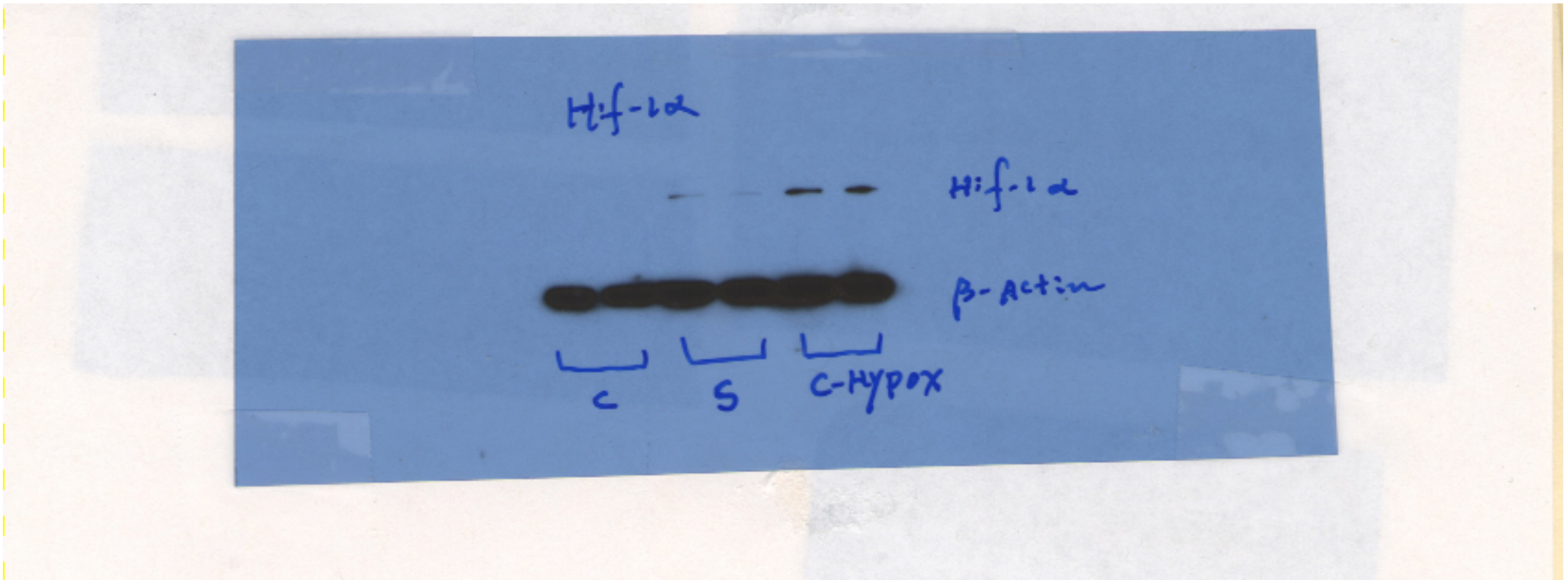


Figure 3E, another different gel, shorter exposure for β-actin

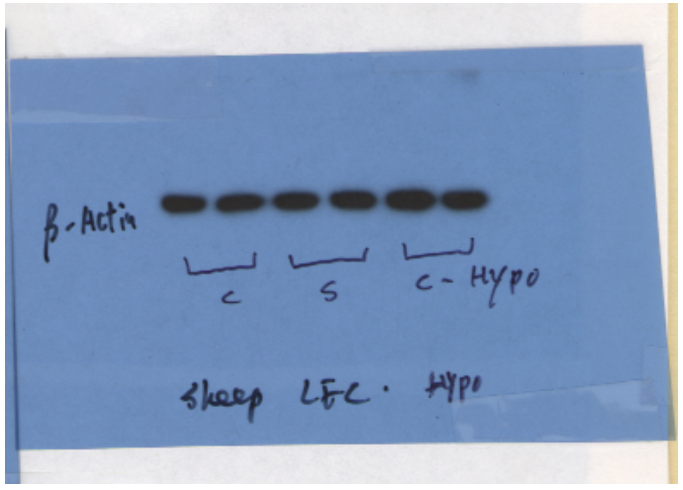


C

Figure 3G, control LECs ± shear uncropped LiCoR image for figure

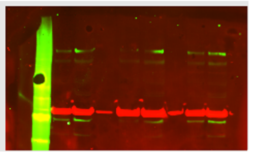

D

Figure 4A, control LECs ± shear ± MQ, uncropped LiCoR images for figure, same samples, parallel gels

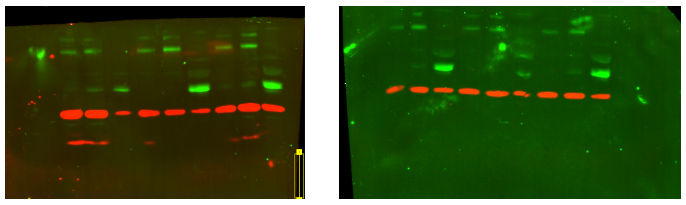

E

Figure 4B, uncropped blots, 20min and 60min exposure

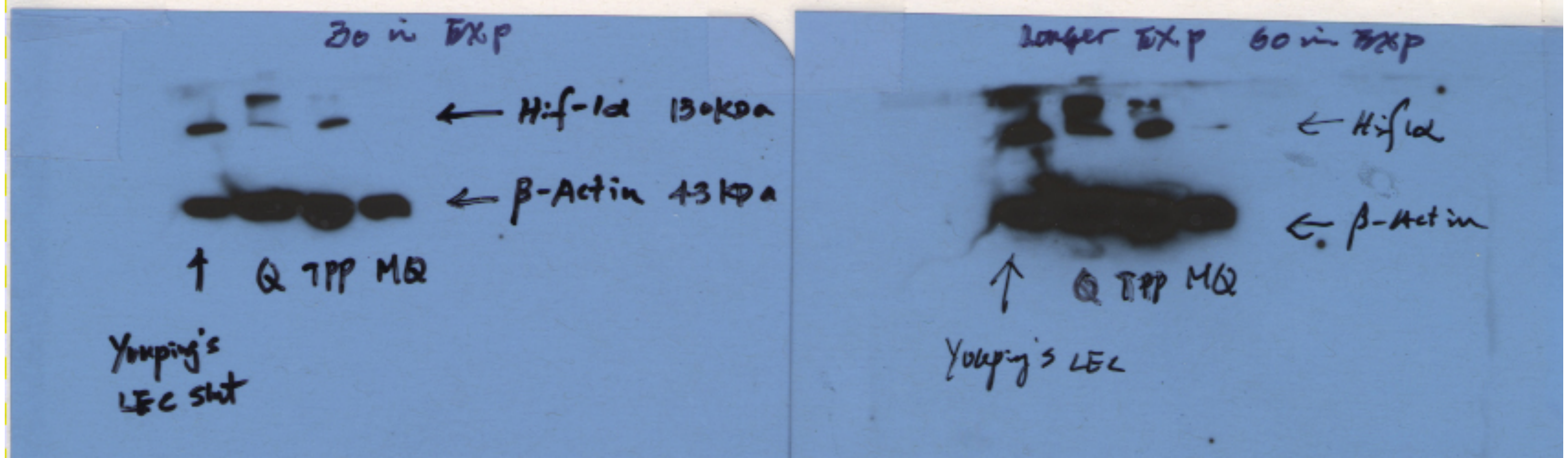

Supplement: Supplementary file 1 — Supplementary Information 1. [file 41598_2020_80882_MOESM1_ESM.pdf]
